# Asthma control and exacerbation risk following SARS-CoV-2 infection in the post-acute COVID-19 phase: a systematic review

**DOI:** 10.1186/s13223-026-01027-z

**Published:** 2026-04-15

**Authors:** Saad Alhumaid, Zainah Sabr, Sara Mohammad Alshehri, Hassan A. Alhashem, Qasim Alnajjad, Dalal Saadoun Alsaadoun, Abeer S. Algrafi, Fatima A. Aborshaid, Ashwag A. Alsaidalani, Sundus Noorsaeed, Dawood Adnan Al Nasser, Rabab Abbas Majzoub, Ola Alkhars, Nourah Al Dossary, Shorooq Shwgi Banjar, Ibtisam Ahmed ALMuhaini, Fatimah Ali Ismail, Zainab Al Alawi, Qasem M. Alalwan

**Affiliations:** 1 School of Pharmacy, University of Tasmania, Hobart, 7000 Australia; 2 Imam Abdulrahman Al-Faisal Hospital, C1 Riyadh Health Cluster, Ministry of Health, Riyadh, 14723 Saudi Arabia; 3 Department of Pediatric, College of Medicine, Imam Mohammed Ibn Saud Islamic University, Riyadh, 11432 Saudi Arabia; 4 Department of Pediatrics, Maternity and Children Hospital, Eastern Health Cluster, Dammam, 32275 Saudi Arabia; 5 Department of Pediatrics, Maternity and Children Hospital, Al-Ahsa Health Cluster, Ministry of Health, Al-Ahsa, 36422 Saudi Arabia; 6 Department of Internal Medicine, College of Medicine, King Faisal University, Al-Ahsa, 31982 Saudi Arabia; 7 Department of Medicine, College of Medicine, Taibah University, Madinah, 42353 Saudi Arabia; 8 Department of Pediatric Allergy, Asthma and Clinical Immunology, Dr. Sulaiman Al-Habib Hospital, Al Khobar, 34423 Saudi Arabia; 9 Department of Pediatrics, Faculty of Medicine, King Abdulaziz University, Jeddah, 21589 Saudi Arabia; 10 Long-term Care Department, Oyun City General Hospital, Al-Ahsa Health Cluster, Ministry of Health, Al-Ahsa, 36312 Saudi Arabia; 11 Department of Pediatrics, College of Medicine, King Faisal University, Al-Ahsa, 31982 Saudi Arabia; 12 Pediatric Department, King Faisal General Hospital, Al-Ahsa Health Cluster, Ministry of Health, Al-Ahsa, 36361 Saudi Arabia; 13 General Surgery Department, King Fahad Hofuf Hospital, Al-Ahsa Health Cluster, Ministry of Health, Al-Ahsa, 36441 Saudi Arabia; 14 Department of Internal Medicine, Faculty of Medicine, King Abdulaziz University, Jeddah, 22252 Saudi Arabia; 15 Cardiology Unit, King Fahad Hofuf Hospital, Al-Ahsa Health Cluster, Ministry of Health, Al-Ahsa, 36441 Saudi Arabia; 16 Pharmacy Department, King Fahad Hofuf Hospital, Al-Ahsa Health Cluster, Ministry of Health, Al-Ahsa, 36441 Saudi Arabia; 17 Division of Allergy and Immunology, College of Medicine, King Faisal University, Al-Ahsa, 31982 Saudi Arabia; 18 Radiology Department, King Fahad Hofuf Hospital, Al-Ahsa Health Cluster, Ministry of Health, Al-Ahsa, 36441 Saudi Arabia

**Keywords:** Asthma, Asthma control, Asthma exacerbation, SARS-CoV-2, COVID-19, Post-acute COVID-19, Long COVID, Respiratory outcomes, Systematic review

## Abstract

**Background:**

The post-acute respiratory consequences of severe acute respiratory syndrome coronavirus 2 (SARS-CoV-2) infection in individuals with asthma remain incompletely understood. While early coronavirus disease 2019 (COVID-19) research focused on acute infection severity, less is known about asthma control and exacerbation risk following recovery, particularly beyond the acute phase.

**Objectives:**

To systematically evaluate asthma control and exacerbation risk following SARS-CoV-2 infection in the post-acute COVID-19 phase among pediatric and adult populations.

**Methods:**

We conducted a systematic review in accordance with Preferred Reporting Items for Systematic Reviews and Meta-Analyses (PRISMA) 2020 guidelines and a prospectively registered PROSPERO protocol (CRD420261290371). PubMed, Embase, CINAHL, Scopus, and Web of Science were searched from 1 January 2020 to 21 January 2026. Observational studies evaluating asthma control or exacerbation outcomes assessed ≥ 4 weeks after confirmed SARS-CoV-2 infection were included. Comparators comprised uninfected asthma controls, within-individual pre–post analyses, or population-level controls. Data were synthesised narratively due to substantial clinical and methodological heterogeneity.

**Results:**

Eleven observational studies involving children and adults with asthma met the eligibility criteria. Evidence regarding post-acute asthma control was heterogeneous. Several studies reported short-term worsening of asthma control following SARS-CoV-2 infection, particularly within the first one to three months, whereas larger longitudinal and registry-based studies generally demonstrated stability or recovery of asthma control over longer follow-up. In contrast, asthma exacerbation risk showed a more consistent pattern, with large population-based studies demonstrating approximately a two- to five-fold increase in post-acute exacerbation risk among individuals hospitalised with COVID-19 (adjusted hazard ratios ranging from 2.78 to 5.12), while risk was lower and more variable among non-hospitalised cases (hazard ratios approximately 1.8–2.1). Findings were more variable and often null among patients with mild infection. No consistent evidence of sustained deterioration in asthma control was observed across longer-term follow-up.

**Conclusion:**

Post-acute asthma outcomes following SARS-CoV-2 infection are heterogeneous and appear to vary according to acute COVID-19 severity, follow-up duration, and study design. While transient worsening of asthma control may occur, particularly after more severe infection, the available evidence does not consistently demonstrate sustained deterioration in asthma control. These findings suggest that post-COVID asthma management may benefit from consideration of infection severity, while highlighting the need for high-quality longitudinal research to better define long-term outcomes.

**Supplementary Information:**

The online version contains supplementary material available at 10.1186/s13223-026-01027-z.

## Background

Asthma is a common chronic respiratory disease affecting over 300 million individuals worldwide and is characterised by variable airflow limitation, airway inflammation, and recurrent exacerbations that contribute substantially to morbidity, healthcare utilisation, and reduced quality of life [1, 2]. Viral respiratory infections are a major trigger of asthma exacerbations and loss of disease control, raising early concerns that infection with severe acute respiratory syndrome coronavirus 2 (SARS-CoV-2) might adversely affect asthma outcomes both during and after acute coronavirus disease 2019 (COVID-19) [3, 4].During the early phases of the COVID-19 pandemic, research largely focused on whether asthma increased susceptibility to SARS-CoV-2 infection or severity of acute COVID-19 outcomes, including hospitalisation, intensive care unit admission, and mortality [3, 5]. While asthma was initially hypothesized to confer increased risk, accumulating evidence suggested that most individuals with asthma were not at substantially higher risk of severe acute COVID-19, particularly when disease was well controlled and inhaled corticosteroid therapy was maintained [6–8]. Consequently, attention shifted toward understanding the longer-term respiratory consequences of SARS-CoV-2 infection.

Post-acute COVID-19 sequelae, often referred to as post-acute COVID-19 syndrome or long COVID, encompass a broad range of persistent symptoms and organ-specific complications that may persist for weeks to months after acute infection [9, 10]. Respiratory manifestations are among the most commonly reported post-acute features, including cough, dyspnoea, and reduced exercise tolerance [9, 11, 12]. In individuals with pre-existing asthma, these post-acute respiratory effects raise important questions regarding potential deterioration in asthma control, increased exacerbation risk, changes in airway inflammation or lung function, and the need for escalation of asthma therapy following recovery from infection.

To date, evidence on post-acute asthma outcomes following SARS-CoV-2 infection has been heterogeneous [9, 12]. Many studies have examined asthma primarily as a baseline comorbidity or risk factor for COVID-19 outcomes, rather than as a condition with its own post-infection disease trajectory [3, 13]. Others have focused on acute or peri-infectious asthma exacerbations, pandemic-related behavioural changes, or long-COVID symptomatology without applying asthma-specific outcome measures or appropriate comparator frameworks [14–16]. As a result, the extent to which SARS-CoV-2 infection influences asthma control and exacerbation risk in the post-acute phase remains incompletely understood.

A systematic synthesis of available evidence focusing specifically on asthma outcomes assessed after recovery from SARS-CoV-2 infection is therefore needed [9, 12]. Clarifying whether post-acute asthma deterioration is transient or sustained, identifying populations at higher risk, and distinguishing infection-related effects from broader pandemic influences are essential to inform post-COVID asthma management and guide future research [9, 10]. This systematic review aims to evaluate asthma control and exacerbation risk following SARS-CoV-2 infection in the post-acute COVID-19 phase, synthesising evidence across pediatric and adult populations using clearly defined post-acute timeframes, asthma-specific outcomes, and appropriate comparator groups.

## Methods

### Study design and reporting framework

This systematic review was conducted and reported in accordance with the Preferred Reporting Items for Systematic Reviews and Meta-Analyses (PRISMA) 2020 guidelines [17], with predefined primary and secondary outcomes specified a priori in the registered protocol. The protocol guided all review methods, including the search strategy, eligibility criteria, study selection, data extraction, and risk of bias assessment, and was registered prospectively in the PROSPERO international prospective register of systematic reviews (CRD420261290371).

## Eligibility criteria

Eligibility criteria were defined using the Population, Exposure, Comparator, Outcomes, and Study design (PICOS) framework (Table 1) [18].

**Table 1:** PICOS eligibility criteria

Domain	Inclusion criteria	Exclusion criteria
Population	Children, adolescents, or adults with physician-diagnosed asthma	Non-asthma populations; studies where asthma-specific outcomes were not reported separately
Exposure	Confirmed SARS-CoV-2 infection (polymerase chain reaction [PCR], antigen testing, serology, or clinically coded COVID-19).	Studies without documented SARS-CoV-2 infection
Post-acute phase	Asthma outcomes assessed ≥ 4 weeks after confirmed acute SARS-CoV-2 infection. The ≥ 4-week threshold was selected to distinguish post-acute outcomes from acute infection–related asthma manifestations, consistent with commonly used definitions of post-acute COVID-19. Where studies defined post-acute outcomes as ≥ 30 days, this was considered equivalent to the ≥ 4-week threshold	Outcomes assessed exclusively during the acute infection phase
Comparator	Asthma patients without SARS-CoV-2 infection; pre–post comparisons within the same individuals anchored to confirmed SARS-CoV-2 infection; population-level historical or contemporaneous controls	Studies without a comparator or longitudinal pre–post assessment
Outcomes	*Primary outcomes* asthma control (e.g., Asthma Control Test [ACT], Childhood Asthma Control Test [cACT], Asthma Control Questionnaire [ACQ], or equivalent) and asthma exacerbations (systemic corticosteroids, emergency department visits, hospitalisation, unscheduled care). *Secondary outcomes* changes in asthma medication use (e.g., step-up therapy, rescue inhaler use), lung function measures (e.g., forced expiratory volume in one second [FEV₁], peak expiratory flow), and asthma-related healthcare utilisation.	Studies reporting only non-asthma respiratory symptoms or outcomes unrelated to asthma control or exacerbation risk
Study design	Observational studies (cohort, case–control, registry-based, longitudinal cross-sectional)	Case reports, small case series (< 10 participants), reviews, editorials, conference abstracts without full text
Publication characteristics	Full-text articles published in peer-reviewed journals	Non-peer-reviewed literature; animal studies

## Study design

We included observational studies, including cohort studies, case–control studies, cross-sectional studies with longitudinal assessment, and registry-based analyses. Case reports, small case series (< 10 participants), narrative reviews, editorials, conference abstracts without full text, and non-human studies were excluded.

The PRISMA 2020 checklist was used (see Supplementary Materials) [17].

## Information sources and search strategy

A comprehensive literature search was conducted in PubMed, Embase, CINAHL (Cumulative Index to Nursing and Allied Health Literature), Scopus, and Web of Science. The search covered studies published from 1 January 2020 to 21 January 2026, which was the date of the final search. The strategy combined controlled vocabulary terms and free-text keywords related to asthma, SARS-CoV-2/COVID-19, and post-acute or long-term outcomes. Reference lists of included studies and relevant reviews were hand-searched to identify additional eligible articles. The full search strategy is provided in Supplementary Table S1.

**Table 2:** Characteristics of studies included in the systematic review

First author (year)	Country	Study design	Data source	Study period	Population (age group)	Sample size (asthma)	SARS-CoV-2 confirmation	Comparator^a^	Post-acute follow-up definition^b^
Abdul-Razzak [23]	Romania	Retrospective cohort study	Single tertiary paediatric hospital medical records (Filantropia Clinical Municipal Hospital, Craiova)	Mar 2020 – Jul 2024	Children with physician-diagnosed asthma (< 18 y)	146 total asthmatic children (79 with SARS-CoV-2 infection; 67 uninfected asthma controls)	RT-PCR or RAT	Asthmatic children without SARS-CoV-2 infection; within-patient pre–post comparisons	Outcomes assessed ≥ 4 weeks after SARS-CoV-2 infection; before–after comparisons of FeNO, lung function, asthma control, and exacerbations during post-acute follow-up
Abdul-Razzak [22]	Romania	Retrospective cohort study	Single tertiary paediatric hospital medical records (Filantropia Clinical Municipal Hospital, Craiova)	Mar 2020 – Jul 2024	Children with physician-diagnosed asthma (0–17 y)	149 total asthmatic children (81 with symptomatic COVID-19; 68 uninfected asthma controls)	PCR or RAT with clinical symptoms	Asthmatic children without SARS-CoV-2 infection	Outcomes assessed ≥ 4 weeks after acute infection; persistent symptoms (e.g., cough > 4 weeks) and lung function changes during post-acute follow-up
Agondi [24]	Brazil	Retrospective cohort study	Single tertiary asthma outpatient clinic (University of São Paulo) medical records	Sept 2020 – Oct 2021	Adults with physician-diagnosed asthma	208 total asthmatic patients (59 with COVID-19; 149 uninfected asthma controls)	PCR and/or serology with compatible clinical symptoms	Asthmatic patients without SARS-CoV-2 infection	Asthma outcomes assessed ≥ 30 days after COVID-19 diagnosis; post-COVID asthma worsening defined by symptom persistence, ≥ 3-point ACT decline, and need for treatment escalation
Chang [25]	Taiwan	Prospective case–control study	Single tertiary pediatric hospital outpatient clinics; population-based asthma cohort for controls (CARE study)	2023 (Omicron-predominant period)	Children with physician-diagnosed asthma (8–15 years)	520 total asthmatic children (336 with SARS-CoV-2 infection; 184 uninfected asthma controls)	RAT (home testing), documented clinically	Asthmatic children without SARS-CoV-2 infection (age-matched controls)	Outcomes assessed 1 month (≈ 4 weeks) after confirmed SARS-CoV-2 infection
Choi [26]	Republic of Korea	Nationwide retrospective cohort study (claims-based, pre–post within-person analysis)	HIRA Service nationwide health insurance claims database	January 2019 – December 2021 (COVID-19 infections identified in 2020; 12-month pre- and post-infection windows)	Adults with asthma aged ≥ 15 years	2,965 asthma patients with confirmed SARS-CoV-2 infection	ICD-10 diagnosis codes (U07.1, U07.2, U09, U09.9) recorded in national claims data	Within-person comparison: 12 months before vs. 12 months after SARS-CoV-2 infection	Outcomes assessed during the 12 months after COVID-19 diagnosis, excluding reinfections; sensitivity analyses excluding events within 14 days
Duong [27]	United States	Retrospective cohort study	Montefiore Health System electronic health records (OMOP CDM)	Mar 2020 – Dec 2023	Children (< 21 y) and adults (≥ 21 y) with pre-existing asthma	10,430 matched (1,898 children; 8,532 adults)	PCR-confirmed SARS-CoV-2 infection	Asthma patients without documented SARS-CoV-2 infection (matched controls)	≥ 30 days after index SARS-CoV-2 test; follow-up up to 3 years
Gaietto [29]	United States	Prospective observational cohort study (registry-based, longitudinal pre–post analysis)	Western Pennsylvania COVID-19 Registry; EHRs from University of Pittsburgh Medical Center Children’s Hospital and affiliated network	Mar 2020 – Jan 2023 (follow-up through Aug 2023)	Children and adolescents with physician-diagnosed asthma (6–21 y)	267 with ACT/cACT data; 196 with spirometry data	PCR or RAT documented in registry/EHR	Within-person pre–post comparison (baseline pre-infection vs. post-infection follow-up)	Outcomes assessed ≥ 4 weeks after confirmed SARS-CoV-2 infection; longitudinal follow-up up to 34 months (median ≈ 11 months)
Gaietto [28]	United States	Prospective case–control observational study (registry-based, longitudinal pre–post analysis)	Western Pennsylvania COVID-19 Registry (cases) and Children’s Hospital of Pittsburgh Asthma Registry (controls); EHRs	Mar 2020 – Aug 2022 (follow-up through Dec 2022)	Children and adolescents with physician-diagnosed asthma (6–21 y)	214 COVID-19 cases (171 with ACT/cACT data; 114 with spirometry); 287 uninfected asthma controls (113 with ACT/cACT; 237 with spirometry)	RT-PCR or rapid antigen test documented in EHR/registry	Asthma patients with SARS-CoV-2 infection vs. uninfected asthma controls; additional within-person pre–post comparisons	Outcomes assessed ≥ 4 weeks after SARS-CoV-2 infection; follow-up within 18 months after acute infection (median ≈ 10–12 months)
Kwok [30]	Hong Kong (China)	Case–control observational study	Single tertiary university hospital asthma clinic (Queen Mary Hospital, Hong Kong); electronic patient records	May 2022 – November 2022 (COVID-19 infection occurred 30–270 days prior to enrolment)	Adults with physician-diagnosed asthma (≥ 18 years)	221 total asthma patients (111 with prior COVID-19; 110 uninfected controls)	Laboratory-confirmed SARS-CoV-2 infection by RT-PCR or RAT, documented in Hospital Authority database	Asthma patients without documented SARS-CoV-2 infection; matched on age, sex, smoking status, asthma severity, and lung function	Asthma outcomes assessed 30–270 days after confirmed SARS-CoV-2 infection, beyond acute illness and mandatory isolation period
Kwok [31]	Hong Kong (China)	Prospective follow-up cohort study	Single tertiary university hospital asthma clinic (Queen Mary Hospital, Hong Kong) electronic patient records	May 2022 – Sep 2023	Adults with physician-diagnosed asthma	189 adults with asthma (41 never infected; 94 prior COVID-19 in 2022 [BA.2]; 54 COVID-19 during follow-up in 2023 [XBB])	Laboratory-confirmed SARS-CoV-2 infection by RT-PCR or rapid antigen test	Never-infected asthma patients and within-cohort comparison by timing of infection (past vs. new COVID-19)	Asthma outcomes assessed ≥ 30 days after recovery; longitudinal follow-up at ~ 6 and 12 months after infection
Lee [32]	Republic of Korea	Nationwide retrospective cohort study (population-based)	Korean NHIS claims database (linked with national SARS-CoV-2 testing and mortality data)	January 2015 – December 2021 (COVID-19 recovery cohort: Oct 2020 – Dec 2021; mortality follow-up to Sept 2022)	Adults with asthma aged ≥ 20 years	21,478 total • COVID-19 cohort: 10,739 • Matched uninfected controls: 10,739	RT-PCR–confirmed SARS-CoV-2 infection (ICD-10 U07.1)	Asthma patients without documented SARS-CoV-2 infection (1:1 propensity score–matched controls)	Index date defined as recovery from acute COVID-19; outcomes assessed ≥ 14–30 days post-recovery, with follow-up up to ~ 2 years

^a^Comparator groups were categorised as uninfected controls (individuals with asthma and no documented SARS-CoV-2 infection), pre–post comparisons within the same individuals (outcomes before vs. after infection), or historical/population controls, as defined by each study

^b^Post-acute COVID-19 phase was defined as outcomes assessed ≥ 4 weeks after confirmed acute SARS-CoV-2 infection, in accordance with commonly used definitions in the literature

ACT, Asthma Control Test; cACT, Childhood Asthma Control Test; CARE, Changhua School Children’s Asthma Screening and Environmental Factors Survey and Health Promotion Project; COVID-19, coronavirus disease 2019; EHRs, electronic health records; FeNO, fractional exhaled nitric oxide; HIRA, Health Insurance Review and Assessment; ICD-10, International Classification of Diseases, 10th Revision; NHIS, National Health Insurance Service; OMOP CDM, Observational Medical Outcomes Partnership Common Data Model; PCR, polymerase chain reaction; RAT, rapid antigen test; RT-PCR, reverse transcription polymerase chain reaction SARS-CoV-2, severe acute respiratory syndrome coronavirus 2

## Study selection

All identified records were imported into reference management software and de-duplicated. Titles and abstracts were screened independently by two reviewers against the eligibility criteria. Full texts of potentially relevant studies were subsequently assessed independently by the same reviewers. Reviewers were not blinded to study authors or journals during screening. Discrepancies at any stage were resolved through discussion or consultation with a third reviewer when required.

**Fig. 1:**
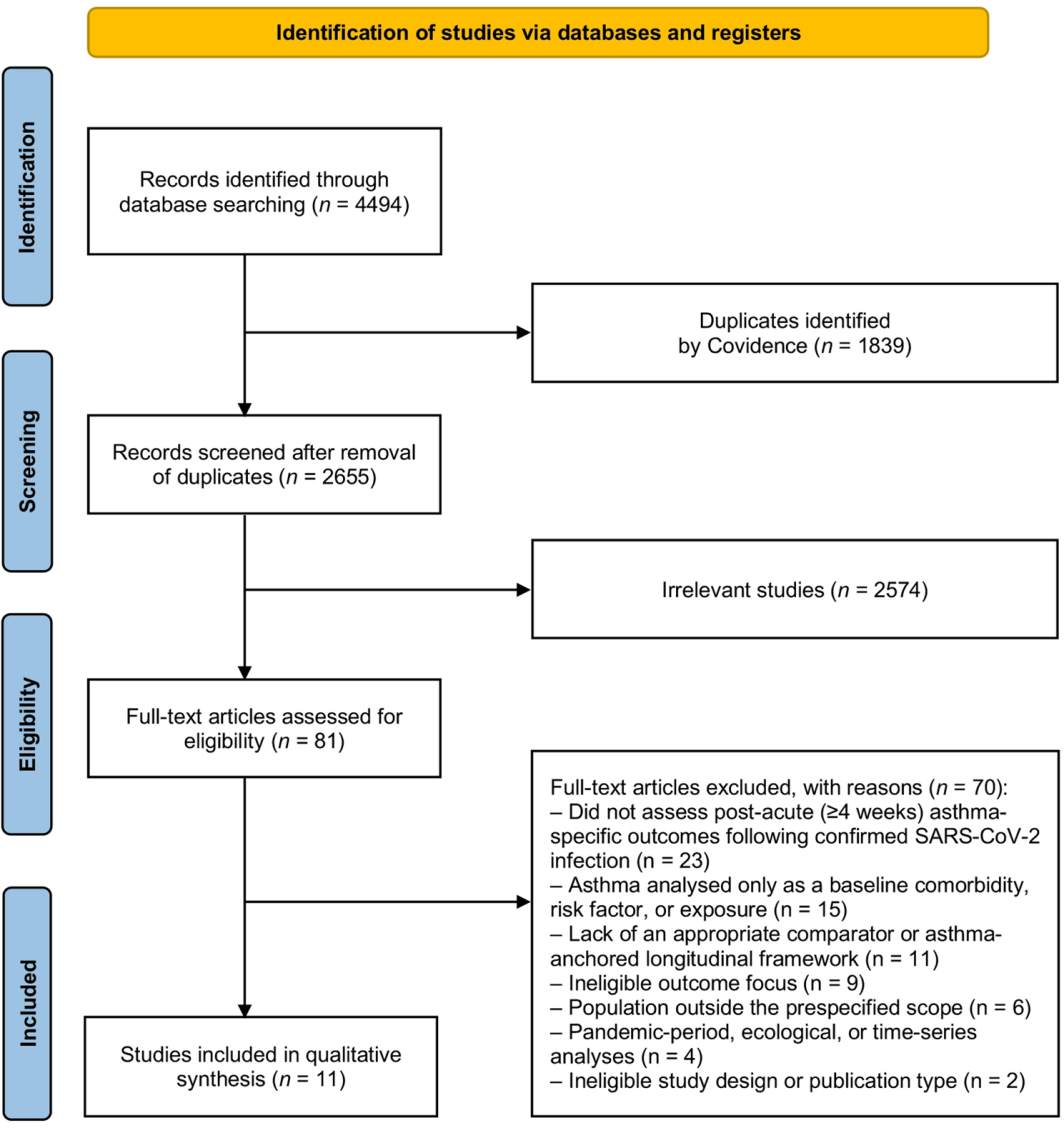
Flow diagram of studies included in the systematic review

The study selection process is summarised using a PRISMA flow diagram (Fig. 1). Reasons for exclusion at the full-text stage were recorded and are presented in Supplementary Table S2.

### Data extraction

Data were extracted independently by two reviewers using a standardised extraction form. Extracted information included study characteristics (author, year, country, study design, and data source), participant characteristics (age, sex, and asthma severity where available), SARS-CoV-2 infection characteristics (confirmation method and timing), definition of the post-acute follow-up period, asthma outcomes and outcome definitions, effect estimates and measures of variability where reported, and adjustment for potential confounding variables. Where multiple effect estimates were reported, those corresponding to the longest post-acute follow-up or the most fully adjusted model were prioritised. Where multiple publications reported results from the same cohort, the most comprehensive or most recent report was used.

## Risk of bias assessment

Risk of bias was assessed independently by two reviewers using the Newcastle–Ottawa Scale (NOS) for cohort and case–control studies [19]. The NOS was applied in accordance with established guidance for observational respiratory outcome studies. The Risk Of Bias In Non-randomized Studies of Interventions (ROBINS-I) tool was not applied given the predominance of cohort designs with clearly defined comparators and outcomes, for which the NOS is commonly used and appropriate [20]. Disagreements were resolved through discussion and consensus.

## Data synthesis

Given anticipated heterogeneity in study design, outcome definitions, follow-up duration, and analytic methods, formal quantitative meta-analysis was not planned a priori. Findings were synthesised narratively, with results summarised by outcome domain (asthma control and exacerbations), age group, and study design. Where appropriate, effect estimates were presented descriptively. Given substantial clinical and methodological heterogeneity in outcome definitions, follow-up duration, and analytic approaches, quantitative meta-analysis was not undertaken.

### Certainty of evidence

The certainty of evidence for key outcomes was assessed qualitatively, considering study limitations, consistency of findings, and precision of effect estimates. Given the observational nature of the included studies and substantial clinical and methodological heterogeneity in study design, populations, outcome definitions, and follow-up duration, a formal Grading of Recommendations Assessment, Development and Evaluation (GRADE) assessment was not undertaken [21]. The absence of a formal GRADE assessment is acknowledged as a limitation and reflects variability in outcome measurement and study design across included studies.

## Results

### Study selection

The database search identified 4,494 potentially relevant records. After removal of duplicates, 2,655 titles and abstracts were screened, of which 2,574 were excluded as clearly irrelevant. Full texts of 81 articles were assessed for eligibility, and 70 studies were excluded after full-text review. The most common reasons for exclusion were the absence of post-acute (≥ 4 weeks) asthma-specific outcomes following confirmed SARS-CoV-2 infection, analysis of asthma solely as a baseline comorbidity or risk factor for COVID-19 outcomes, and lack of appropriate comparators or asthma-anchored pre–post analyses. Additional exclusions included studies focused exclusively on acute COVID-19 severity, long-COVID symptomatology without asthma-specific outcomes, mixed or non-asthma populations without stratified analyses, ineligible study designs, pandemic-period ecological analyses, non-peer-reviewed reports, and overlapping evidence. The study selection process is summarised in Fig. 1, with detailed reasons for exclusion provided in Supplementary Table S2.

A total of 11 observational studies met the eligibility criteria and were included in the qualitative synthesis [22–32].

### Characteristics of included studies

The characteristics of included studies are summarised in Table 2. The 11 studies comprised retrospective and prospective cohort studies, case–control studies, and registry-based analyses conducted across Europe, North America, East Asia, and South America. Study populations included both children and adults with physician-diagnosed asthma, with sample sizes ranging from 146 to over 21,000 individuals. SARS-CoV-2 infection was confirmed using reverse transcription polymerase chain reaction (RT-PCR), rapid antigen testing, serology, or validated diagnostic codes. Comparators included uninfected asthma controls, within-person pre–post comparisons, or combinations of both. Post-acute follow-up was defined as outcomes assessed ≥ 4 weeks after acute infection, with follow-up durations ranging from approximately 1 month to 3 years.

### Asthma control outcomes following SARS-CoV-2 infection

Asthma control outcomes are summarised in Table 3. Among pediatric populations, several studies reported worse asthma control following SARS-CoV-2 infection, including higher proportions of partially controlled or uncontrolled asthma and lower Childhood Asthma Control Test (cACT) scores compared with uninfected asthma controls [22, 23, 25]. These findings were most pronounced in studies with short-term post-acute follow-up (approximately 1 month) and in cohorts where controller therapy was modified following infection [22, 25].

**Table 3:** Asthma control outcomes following SARS-CoV-2 infection in the post-acute phase

First author (year)	Age group	Asthma severity	Control measure (ACT / ACQ / cACT)	Follow-up duration	Comparison	Effect estimate	95% CI / SD	Adjusted covariates
Abdul-Razzak [23]	Children (0–17 y)	Mixed severity (GINA steps 1–5; controller therapy in use)	GINA asthma control categories (well-controlled vs. partially controlled)^c^	Post-acute follow-up ≥ 4 weeks after SARS-CoV-2 infection; serial clinical visits during Mar 2020–Jul 2024	Asthmatic children with prior SARS-CoV-2 infection vs. asthmatic children without infection; additional within-cohort analyses by parental dose adjustment	Higher proportion of partially controlled asthma among post-COVID-19 children, particularly in those with parental step-down of controller therapy	NR	NR (unadjusted comparisons)
Abdul-Razzak [22]	Children (0–17 y)	Mixed severity (allergic and non-allergic phenotypes; controller therapy in use)	GINA asthma control categories (well controlled vs. partially controlled)^c^	Post-acute follow-up ≥ 4 weeks after SARS-CoV-2 infection; clinical visits during Mar 2020–Jul 2024	Asthmatic children with prior SARS-CoV-2 infection vs. asthmatic children without infection	Higher proportion of partially controlled asthma among post-COVID-19 children	NR	NR (unadjusted comparisons)
Agondi [24]	Adults (mean age 52.8 y)	Predominantly severe asthma (GINA steps 4–5 in 73%)	ACT^a^	Post-acute assessment ≥ 30 days after COVID-19; follow-up Sept 2020–Oct 2021	Asthmatic patients with SARS-CoV-2 infection vs. asthmatic patients without infection	Asthma worsening in 33.9% vs. 11.4% of patients^d^	NR	NR (unadjusted comparisons)
Chang [25]	Children (8–15 years)	Mixed severity; asthma classified by cACT categories (controlled, partially controlled, uncontrolled)	cACT^b^	Post-acute assessment 1 month (≈ 4 weeks) after SARS-CoV-2 infection	Asthmatic children with prior SARS-CoV-2 infection vs. asthmatic children without SARS-CoV-2 infection	Lower asthma control scores in post-COVID-19 children	Median cACT: 25 (IQR 23–27) in post-COVID-19 vs. 27 (IQR 24.3–27) in uninfected controls; *p* < 0.001	Age, sex, height, weight, FEV₁/FVC, COVID-19 vaccination status, FeNO levels (multivariable linear regression)
Gaietto [29]	Children and adolescents (6–21 y)	Mixed severity (intermittent to severe persistent; NAEPP-defined)	ACTᵃ / cACTᵇ (combined as transformed ACTS score)	Post-acute assessment ≥ 4 weeks after SARS-CoV-2 infection; longitudinal follow-up up to 34 months (median 11.4 months)	Within-person pre–post comparison (baseline pre-infection vs. post-infection follow-up)	No significant change in asthma symptom control after SARS-CoV-2 infection	Mean change in ACTS: 0.0 ± 4.7; *p* = 0.60	Age, sex, race, baseline asthma severity (adjusted mixed-effects models with linear spline)
Gaietto [28]	Children and adolescents (6–21 y)	Mixed severity (intermittent to severe persistent; NAEPP-defined)	ACTᵃ / cACTᵇ (combined as transformed ACTS score)	Post-acute assessment ≥ 4 weeks after SARS-CoV-2 infection; follow-up within 18 months (median ~ 10–12 months)	Asthma patients with SARS-CoV-2 infection vs. uninfected asthma controls; additional within-person pre–post comparisons	No significant difference in asthma symptom control after SARS-CoV-2 infection	Mean change in ACTS: 0.0 ± 4.6; *p* = 0.97 (cases). Follow-up ACTS similar in cases vs. controls	Age, sex, race, baseline asthma severity, time interval between COVID-19 and follow-up (multivariable models)
Kwok [30]	Adults (≥ 18 years; mean age 58 ± 16 y)	Mixed severity (GINA steps 1–5; majority steps 3–5)	ACT^a^	Post-acute assessment 30–270 days after SARS-CoV-2 infection; mean 47 ± 57 days from COVID-19 diagnosis to enrolment	Asthma patients with prior mild–moderate COVID-19 vs. matched asthma patients without COVID-19	≥ 3-point ACT decline more frequent after COVID-19^e^	Adjusted OR = 3.11 (95% CI 1.39–6.96) for ≥ 3-point ACT decline	Age, sex, smoking status, baseline FEV₁ (% predicted), COVID-19 vaccination status, baseline ACT score, baseline GINA step
Kwok [31]	Adults (mean age 57.7 y)	Mixed severity (GINA steps 1–5; majority steps 3–4)	ACT^a^	Post-acute follow-up at ~ 6 and 12 months after recovery; May 2022–Sep 2023	Never-infected asthma vs. past COVID-19 (BA.2) vs. new COVID-19 during follow-up (XBB)	No significant difference in ACT change between groups; ACT change − 0.34 ± 3.7 (no COVID-19), 0.0 ± 5.0 (past COVID-19), − 0.17 ± 4.5 (new COVID-19); worsening (ACT change ≥ 3) in 24.4%, 25.5%, and 22.2%, respectively	NR	Age, sex, smoking status, baseline FEV₁ (% predicted), vaccination status, baseline ACT score, baseline GINA step
Lee [32]	Adults (≥ 20 years)	Indirectly defined using treatment steps and prior severe exacerbation history (no control score stratification)	NR	Median 87 days (exacerbations); median 360 days (mortality)	COVID-19 asthma cohort vs. propensity-score–matched uninfected asthma controls	NA	NA	NA (no asthma control outcome)

^a^Asthma Control Test (ACT) scores ≥ 20 were considered indicative of well-controlled asthma, in accordance with established validation studies

^b^Childhood Asthma Control Test (cACT) scores ≥ 20 were considered indicative of well-controlled asthma

Several included studies evaluated post-acute asthma outcomes using clinical exacerbation or healthcare utilisation endpoints without reporting validated asthma control scores (ACT, cACT, or ACQ); such studies are not represented in this table

^c^Asthma control was assessed using GINA guideline–based clinical evaluation rather than validated questionnaire scores (ACT, cACT, or ACQ). The study reported statistically significant differences in asthma control distribution between SARS-CoV-2–infected and uninfected children and strong associations between parental step-down of therapy and partially controlled asthma (*χ²* tests, *p* < 0.0005), but did not report adjusted effect estimates

^d^In Agondi et al. [24], asthma worsening was defined as a ≥ 3-point decline in ACT score from baseline, consistent with the minimally important difference

^e^In Kwok et al. [30], post-acute SARS-CoV-2 infection was additionally associated with higher odds of uncontrolled asthma (ACT ≤ 15; adjusted OR 5.51, 95% CI 1.06–28.60) and a greater mean decline in ACT score (− 2.47 ± 4.96 vs. + 0.40 ± 3.46 in uninfected controls)

ACQ, Asthma Control Questionnaire; ACT, Asthma Control Test; ACTS, transformed ACT score and cACT score (collectively); cACT, Childhood Asthma Control Test; CI, confidence interval; COVID-19, coronavirus disease 2019; FeNO, fractional exhaled nitric oxide; FEV₁, forced expiratory volume in one second; FVC, forced vital capacity; GINA, Global Initiative for Asthma; IQR, interquartile range; NA, not applicable; NAEPP, National Asthma Education and Prevention Program; NR, not reported; OR, odds ratio; SD, standard deviation; SARS-CoV-2, severe acute respiratory syndrome coronavirus 2

In contrast, larger registry-based and longitudinal pediatric studies using within-person pre–post comparisons did not demonstrate sustained deterioration in asthma control over longer follow-up periods, with mean Asthma Control Test (ACT)/cACT scores remaining stable and many patients demonstrating partial or complete recovery over time [28, 29].

Among adults, evidence was mixed. Some studies reported increased odds of clinically meaningful worsening in asthma control (e.g., ≥ 3-point decline in ACT score) following mild-to-moderate COVID-19 [24, 30], whereas others observed no significant differences in asthma control trajectories when comparing infected and uninfected asthma patients or when stratifying by Omicron subvariant exposure [29, 31].

### Asthma exacerbation risk following SARS-CoV-2 infection

Asthma exacerbation outcomes are summarised in Table 4. In children and adults, several studies reported increased exacerbation risk following SARS-CoV-2 infection, particularly among patients with more severe acute COVID-19 [27, 32]. Large population-based and electronic health record–based cohort studies demonstrated higher rates of post-acute asthma exacerbations among individuals hospitalised with COVID-19 compared with uninfected asthma controls, with adjusted hazard ratios indicating approximately a two- to five-fold increase in risk [27, 32]. Elevated exacerbation risk was also observed, to a lesser extent, among non-hospitalised COVID-19 cases in some cohorts [27].

**Table 4:** Asthma exacerbation risk following SARS-CoV-2 infection in the post-acute phase

First author (year)	Age group	Exacerbation definition^a^	Follow-up duration^b^	Comparison	Effect measure (RR/OR/HR)^c^	Effect estimate	95% CI	Adjusted covariates
Abdul-Razzak [23]	Children (0–17 y)	Asthma exacerbations defined as the number of clinically recorded exacerbations per year during post-acute follow-up	Post-acute follow-up ≥ 4 weeks after SARS-CoV-2 infection; clinical follow-ups during Mar 2020–Jul 2024	Asthmatic children with prior SARS-CoV-2 infection vs. asthmatic children without infection	NR	Higher frequency of asthma exacerbations among post-COVID-19 children; exacerbations more common with parental step-down of controller therapy^d^	NR	None (unadjusted comparisons)
Abdul-Razzak [22]	Children (0–17 y)	Asthma exacerbations defined by increased frequency of clinically recorded exacerbations requiring medical management	Post-acute follow-up ≥ 4 weeks after SARS-CoV-2 infection; clinical monitoring during Mar 2020–Jul 2024	Asthmatic children with prior SARS-CoV-2 infection vs. asthmatic children without infection	NR	Higher exacerbation frequency among post-COVID-19 children	NR	None (unadjusted comparisons)
Agondi [24]	Adults (mean age 52.8 y)	Asthma worsening defined as persistence of asthma symptoms ≥ 30 days after COVID-19, associated with ≥ 3-point decline in ACT score and need for step-up of maintenance therapy	Post-acute follow-up ≥ 30 days after SARS-CoV-2 infection; follow-up Sept 2020–Oct 2021	Asthmatic patients with SARS-CoV-2 infection vs. asthmatic patients without infection	OR (unadjusted)	33.9% vs. 11.4% (COVID-19 vs. no COVID-19); *p* < 0.001	NR	None (unadjusted comparisons)
Choi [26]	Adults (≥ 15 y; mean age 56.5 y)	Claims-based exacerbation definitions^e^	12 months post-COVID-19 diagnosis (compared with 12 months pre-COVID-19)	Post-COVID-19 vs. pre-COVID-19 (within-person comparison)	IRR (binomial mixed model)	Moderate exacerbations: 0.848; Moderate-to-severe exacerbations: 0.912; Severe exacerbations: 1.220	Moderate: 0.815–0.882; Moderate-to-severe: 0.887–0.938; Severe: 1.151–1.295	Nationwide claims-based recurrent-event model; sensitivity analyses excluding early events (≤ 14 days) and excluding COPD
Duong [27]	Children (< 21 y)	ICD-10–defined asthma exacerbations (recurrent events); sensitivity analysis requiring systemic corticosteroid treatment	Up to 3 years	Hospitalised COVID-19 vs. uninfected asthma controls	HR	3.29	2.27–4.76	Allergic rhinitis, GERD, eczema, COPD, social determinants of health, COVID-19 wave
Duong [27]	Children (< 21 y)	Same as above	Up to 3 years	Non-hospitalised COVID-19 vs. uninfected asthma controls	HR	1.82	1.51–2.21	Same as above
Duong [27]	Adults (≥ 21 y)	ICD-10–defined asthma exacerbations (recurrent events); sensitivity analysis requiring systemic corticosteroid treatment	Up to 3 years	Hospitalised COVID-19 vs. uninfected asthma controls	HR	2.78	2.29–3.38	Obesity, allergic rhinitis, GERD, COPD, diabetes, depression/anxiety, insurance status, social determinants of health, COVID-19 wave
Duong [27]	Adults (≥ 21 y)	Same as above	Up to 3 years	Non-hospitalised COVID-19 vs. uninfected asthma controls	HR	2.13	1.73–2.62	Same as above
Kwok [30]	Adults (≥ 18 years; mean age 58 ± 16 y)	Asthma exacerbation requiring medical attendance and/or OCS prescription	Post-acute assessment 30–270 days after SARS-CoV-2 infection; mean 47 ± 57 days from COVID-19 diagnosis to enrolment	Asthma patients with prior mild–moderate COVID-19 vs. matched asthma patients without COVID-19	OR (unadjusted logistic regression)	No statistically significant increase in post-acute asthma exacerbations^f^	Medical attendance for exacerbation: OR 1.08 (95% CI 0.47–2.49); OCS-treated exacerbation: OR 1.30 (95% CI 0.47–3.62)	None (exacerbation outcomes not included in multivariable models)
Kwok [31]	Adults (mean age 57.7 y)	Asthma exacerbation defined as episodes requiring OCS, hospitalisation, or ICU/MV	Post-acute follow-up from Nov 2022 to Sep 2023 (up to ~ 12 months after recovery)	Never-infected asthma vs. past COVID-19 (BA.2) vs. new COVID-19 during follow-up (XBB)	OR (logistic regression)	Any exacerbation: 0% (no COVID-19) vs. 8.5% (past COVID-19) vs. 3.7% (new COVID-19); *p* = 0.11	NR	Age, sex, smoking status, baseline FEV₁ (% predicted), COVID-19 vaccination status, baseline ACT score, baseline GINA step
Lee [32]	Adults (≥ 20 y)	Severe asthma exacerbation defined as emergency room visit or hospitalisation with concurrent systemic corticosteroid use	Median 87 days (range 15–448 days) after recovery from COVID-19	COVID-19 asthma cohort vs. propensity-score–matched uninfected asthma controls	HR	1.57	1.06–2.32	Age, sex, BMI, smoking status, alcohol consumption, physical activity, income status, residential area, prior severe exacerbation history, asthma treatment step, allergic rhinitis, HTN, DM, CKD, dyslipidaemia, COPD
Lee [32]	Adults (≥ 20 y)	Same as above	Median 87 days (range 15–448 days)	Severe COVID-19 vs. propensity-score–matched uninfected asthma controls	HR	5.12	3.27–8.01	Same as above
Lee [32]	Adults (≥ 20 y)	Same as above	Median 87 days (range 15–448 days)	Non-severe COVID-19 vs. propensity-score–matched uninfected asthma controls	HR	0.83	0.51–1.34	Same as above

^a^Asthma exacerbations were defined according to individual study criteria and could include systemic corticosteroid use, emergency department visits, asthma-related hospitalisation, or unscheduled asthma-related healthcare encounters

^b^The post-acute COVID-19 phase was defined as outcomes assessed ≥ 30 days after the index SARS-CoV-2 PCR test; sensitivity analyses using stricter exacerbation definitions (systemic corticosteroid–treated events only) and excluding SARS-CoV-2 reinfections yielded consistent results

^c^Effect estimates are adjusted hazard ratios (aHRs) derived from Andersen–Gill recurrent-event models

^d^In Abdul-Razzak et al. (2025), asthma exacerbations were summarised as the number of recurrent exacerbations per year during follow-up. Statistically significant associations were observed between SARS-CoV-2 infection and higher exacerbation frequency, as well as between parental step-down of controller therapy and increased exacerbations (*χ²* tests, *p* < 0.0005); adjusted effect estimates or relative risk measures were not reported

^e^In claims-based studies, asthma exacerbations were identified using ICD-10 diagnosis codes in combination with prescriptions for systemic corticosteroids and/or antibiotics; moderate and severe events were analysed separately using recurrent-event models

^f^In Kwok et al. [30], asthma exacerbation outcomes (medical attendance and systemic corticosteroid use) were analysed descriptively and using unadjusted logistic regression; no statistically significant differences were observed between post-COVID-19 and uninfected asthma patients

ACT, Asthma Control Test; BMI, body mass index; CI, confidence interval; CKD, chronic kidney disease; COPD, chronic obstructive pulmonary disease; COVID-19, coronavirus disease 2019; DM, diabetes mellitus; FEV₁, forced expiratory volume in one second; GERD, gastroesophageal reflux disease; GINA, Global Initiative for Asthma; HR, hazard ratio; HTN, hypertension; ICD-10, International Classification of Diseases, 10th Revision; ICU, intensive care unit; IRR, incidence rate ratio; MV, mechanical ventilation; NR, not reported; OCS, oral corticosteroids; OR, odds ratio; PCR, polymerase chain reaction; RR, risk ratio; SARS-CoV-2, severe acute respiratory syndrome coronavirus 2

Conversely, some studies of mild or moderate COVID-19 reported no statistically significant increase in post-acute asthma exacerbations, particularly when outcomes were assessed over shorter follow-up intervals or when analyses relied on unadjusted models [26, 30, 31]. Overall, the magnitude of exacerbation risk appeared to vary by COVID-19 severity, age group, outcome definition, and analytic approach.

### Additional asthma-related outcomes

Additional asthma-related outcomes are summarised in Table 5. Several pediatric studies reported transient post-acute changes in lung function and airway inflammation following SARS-CoV-2 infection, including reductions in forced expiratory volume in one second or forced expiratory volume in one second/forced vital capacity ratios and increases in fractional exhaled nitric oxide [22, 23, 25]. Persistent respiratory symptoms, such as cough lasting longer than four weeks, were also reported in some cohorts [22–24].

**Table 5:** Additional asthma-related outcomes following SARS-CoV-2 infection in the post-acute phase^a^

First author (year)	Outcome domain	Outcome definition	Follow-up duration	Effect estimate	Direction of effect
Abdul-Razzak [23]	Lung function	Spirometric parameters (FVC, FEV₁, PEF, FEF25–75) assessed before vs. after SARS-CoV-2 infection	Post-acute follow-up ≥ 4 weeks after infection; serial assessments during Mar 2020–Jul 2024	Median FEV₁ decreased from 0.82 to 0.78; median FEF25–75 decreased from 0.78 to 0.70 in infected children (*p* < 0.0005)	Worsened lung function after SARS-CoV-2 infection
Abdul-Razzak [23]	Airway inflammation	Fractional exhaled nitric oxide (FeNO, ppb) measured before vs. after infection	Same as above	Median FeNO increased from 22 to 30 in infected children (*p* < 0.0005)	Increased airway inflammation post-COVID-19
Abdul-Razzak [23]	Persistent respiratory symptoms	Persistent cough > 4 weeks after SARS-CoV-2 infection	Post-acute follow-up ≥ 4 weeks	Higher FeNO levels in children with cough persisting > 4 weeks (median 36.5 ppb vs. 22.5 ppb; *p* < 0.0005)	Persistent symptoms associated with inflammation
Abdul-Razzak [23]	Treatment modification	Parental self-management of controller therapy (step-down, same, step-up)	Same as above	Step-down associated with higher FeNO (median 39.5 ppb) and lower FEV₁ (median 0.78) vs. same or step-up (*p* < 0.05)	Treatment step-down linked to worse outcomes
Abdul-Razzak [23]	Asthma control status	GINA asthma control categories (well-controlled vs. partially controlled)	Same as above	80.95% of children with parental step-down had partially controlled asthma vs. 31.82% with unchanged therapy (*p* < 0.0005)	Reduced asthma control post-COVID-19
Abdul-Razzak [23]	Healthcare utilisation (exacerbation burden)	Number of asthma exacerbations per year	Same as above	Higher exacerbation frequency in children with parental step-down; FeNO increased with increasing exacerbation count (*p* < 0.0005)	Greater exacerbation burden
Abdul-Razzak [22]	Lung function	Spirometric parameters (FEV₁, FEV₁/FVC, FEF25–75, PEF) assessed during post-acute follow-up	Post-acute follow-up ≥ 4 weeks after SARS-CoV-2 infection; clinical visits during Mar 2020–Jul 2024	NR	Reduced lung function (lower FEV₁, FEV₁/FVC, FEF25–75 in infected vs. uninfected children)
Abdul-Razzak [22]	Airway inflammation	Fractional exhaled nitric oxide (FeNO, ppb)	Same as above	NR	Increased airway inflammation (higher FeNO in post-COVID-19 children, especially with cough > 4 weeks)
Abdul-Razzak [22]	Persistent respiratory symptoms	Persistent cough lasting > 4 weeks after acute SARS-CoV-2 infection	Same as above	NR	Higher prevalence of persistent cough among post-COVID-19 children
Abdul-Razzak [22]	Treatment modification / adherence	Changes in controller therapy adherence after infection	Same as above	NR	Reduced adherence / treatment modification more frequent after SARS-CoV-2 infection
Agondi [24]	Asthma medication escalation	Need for step-up in maintenance asthma therapy following SARS-CoV-2 infection	Post-acute follow-up ≥ 30 days after COVID-19; mean duration of treatment escalation ~ 6.1 months	NR	Increased need for treatment escalation among post-COVID-19 asthma patients
Agondi [24]	Persistent asthma symptoms	Persistence of asthma symptoms ≥ 30 days after SARS-CoV-2 infection	Same as above	NR	Persistent respiratory symptoms more frequent after COVID-19
Agondi [24]	Duration of asthma worsening	Duration of worsened asthma control requiring intensified therapy	Same as above	Mean duration ~ 6.14 months	Prolonged asthma worsening following SARS-CoV-2 infection
Agondi [24]	Healthcare utilisation	Hospitalisation for acute COVID-19 (oxygen therapy required)	Acute phase with post-acute follow-up	18.6% hospitalised; no ICU or mechanical ventilation	Limited severe healthcare utilisation, despite post-acute asthma worsening
Chang [25]	Airway inflammation	FeNO (ppb), measured in accordance with ATS guidelines	Post-acute assessment 1 month (≈ 4 weeks) after confirmed SARS-CoV-2 infection	Median FeNO 16.1 ppb (IQR 11.0–27.5) in post-COVID-19 children vs. 9.0 ppb (IQR 2.0–22.1) in uninfected asthma controls; *p* < 0.001	Increased airway inflammation after SARS-CoV-2 infection
Chang [25]	Lung function	Spirometric parameters (FEV₁, FVC, FEV₁/FVC, FEF25–75; % predicted)	Post-acute assessment 1 month after infection	Lower FEV₁/FVC in post-COVID-19 asthma group compared with uninfected controls (*p* < 0.001); no significant difference in FEV₁ or FVC	Mild post-acute impairment in airflow ratios
Chang [25]	Asthma control status (distribution)	Proportion of controlled, partially controlled, and uncontrolled asthma based on cACT categories	1 month post-infection	Higher proportion of partially controlled and uncontrolled asthma among post-COVID-19 children compared with uninfected asthma controls (*p* < 0.001)	Worse asthma control distribution after SARS-CoV-2 infection
Gaietto [29]	Lung function	Spirometric parameters (FEV₁ % predicted, FVC % predicted, FEV₁/FVC) assessed before vs. after SARS-CoV-2 infection	Post-acute assessment ≥ 4 weeks after infection; longitudinal follow-up up to 34 months (median 11.4 months)	No significant difference between baseline and final follow-up (mean change in FEV₁ %pred: 0.0 ± 11.5; *p* = 0.99; FVC and FEV₁/FVC also NS)	No sustained post-acute impairment in lung function
Gaietto [29]	Asthma control trajectory / recovery	Longitudinal recovery of asthma symptom control among children with initial post-COVID worsening (ACTS decline ≥ 3 points at first follow-up)	Same as above	38% fully recovered to baseline ACTS at final follow-up; 58% achieved partial recovery at any subsequent follow-up; final ACTS remained in controlled range (> 19) even without full recovery	Transient worsening with substantial recovery over time
Gaietto [29]	Risk modifiers	Association of obesity with impaired recovery of asthma control after SARS-CoV-2 infection	Same as above	Obesity associated with hindered recovery of asthma symptom control (*p* = 0.04)	Obesity linked to poorer post-acute recovery
Gaietto [29]	Variant-specific effects	Asthma control and lung function outcomes stratified by SARS-CoV-2 variant wave (Pre-Delta, Delta, Omicron)	Same as above	No significant differences in ACTS or spirometry changes across variant waves (all *p* > 0.20)	No variant-specific post-acute effect detected
Gaietto [28]	Lung function	Spirometric parameters (FEV₁ % predicted, FVC % predicted, FEV₁/FVC, FEF25–75% predicted) assessed before vs. after SARS-CoV-2 infection	Post-acute assessment ≥ 4 weeks after infection; follow-up within 18 months after acute COVID-19 (median ~ 10–12 months)	No significant change in lung function after SARS-CoV-2 infection (e.g. mean ΔFEV₁ −0.6 ± 9.2%pred, *p* = 0.47; ΔFVC − 0.6 ± 8.6%pred, *p* = 0.43; ΔFEV₁/FVC − 0.5 ± 6.3, *p* = 0.43; ΔFEF25–75 − 0.9 ± 19.8%pred, *p* = 0.62)	No sustained post-acute impairment in lung function
Gaietto [28]	Asthma control trajectory	Proportion of children with clinically meaningful worsening of asthma control (ACTS decrease ≥ 3 points) at post-acute follow-up	Same as above	17.5% of infected children had worse asthma control at follow-up; proportion similar to uninfected asthma controls (9.7%; *p* = 0.07)	Transient worsening in a minority; no excess post-acute burden vs. controls
Gaietto [28]	Risk modifiers	Association between acute COVID-19 asthma exacerbation and later asthma control or lung function	Same as above	Acute asthma exacerbation during COVID-19 associated with higher odds of worse asthma control in unadjusted analysis (OR 2.47, 95% CI 1.04–5.87) but not after multivariable adjustment; no association with post-acute lung function decline	Acute-phase severity may influence short-term control but not sustained post-acute outcomes
Kwok [30]	Treatment escalation	Escalation of asthma maintenance therapy by ≥ 1 GINA step after recovery from SARS-CoV-2 infection	Post-acute assessment 30–270 days after SARS-CoV-2 infection; mean 47 ± 57 days from diagnosis to enrolment	Adjusted OR = 4.73	Increased need for treatment escalation after COVID-19
Kwok [30]	Asthma control status	Proportion of patients with uncontrolled asthma (ACT ≤ 15) at post-acute follow-up	Same as above	Adjusted OR = 5.51 (95% CI 1.06–28.6)	Higher likelihood of uncontrolled asthma after COVID-19
Kwok [30]	Change in asthma control over time	Mean change in ACT score from prior visit to post-acute follow-up	Same as above	−2.47 ± 4.96 (COVID-19) vs. + 0.4 ± 3.46 (uninfected controls)	Greater decline in asthma control after COVID-19
Kwok [31]	Treatment escalation	Increase in asthma maintenance therapy by ≥ 1 GINA step	Post-acute follow-up Nov 2022–Sep 2023 (up to ~ 12 months after recovery)	7.3% (no COVID-19) vs. 16.0% (past COVID-19 BA.2) vs. 3.7% (new COVID-19 XBB); adjusted OR 3.36 (past COVID-19 vs. no COVID-19), *p* = 0.17	No statistically significant increase in treatment escalation after XBB infection; numerically higher escalation after BA.2
Kwok [31]	Asthma exacerbation severity	Severity of post-acute asthma exacerbations (outpatient OCS vs. hospitalisation)	Same as above	Past COVID-19: 8.5% any exacerbation (6 outpatient OCS, 2 hospitalised); New COVID-19: 3.7% outpatient OCS only; No COVID-19: 0%	Exacerbations were infrequent and predominantly mild, especially after XBB
Kwok [31]	Longitudinal recovery of asthma control	Change in ACT score over extended follow-up among patients with prior post-COVID worsening	Same as above	Mean ACT change from prior study to final follow-up: −0.34 ± 3.7 (no COVID-19), 0.0 ± 5.0 (past COVID-19), − 0.17 ± 4.5 (new COVID-19); *p* = 0.94	Improvement / normalization of asthma control over time after earlier BA.2-associated worsening
Kwok [31]	Variant-specific post-COVID effects	Comparison of asthma outcomes after Omicron BA.2 vs. XBB infection	Same as above	No excess worsening of ACT or exacerbations after XBB compared with BA.2	Later Omicron variants (XBB) associated with milder post-acute asthma impact
Lee [32]	Mortality	All-cause mortality following recovery from SARS-CoV-2 infection	Median 360 days (range 15–721 days) after recovery from COVID-19	HR = 1.75	Increased risk of death among adults with asthma after COVID-19 compared with uninfected controls
Lee [32]	Mortality	All-cause mortality following severe COVID-19 (hospitalisation with oxygen therapy, ICU admission, MV, or ECMO)	Median 360 days	HR = 7.31 (95% CI 5.41–9.88)	Markedly increased mortality risk following severe COVID-19
Lee [32]	Mortality	All-cause mortality following non-severe COVID-19	Median 360 days	0.73 (95% CI 0.51–1.04)	No statistically significant increase in mortality risk

^a^Additional outcomes were prespecified secondary outcomes but were variably defined and inconsistently reported across studies; results are presented descriptively

%pred, percent predicted; ACT, Asthma Control Test; ACTS, transformed ACT score and cACT score (collectively); ATS, American Thoracic Society; CI, confidence interval; COVID-19, coronavirus disease 2019; ECMO, extracorporeal membrane oxygenation; FeNO, fractional exhaled nitric oxide; FEF25–75, forced expiratory flow between 25% and 75% of forced vital capacity; FEV₁, forced expiratory volume in one second; FVC, forced vital capacity; GINA, Global Initiative for Asthma; HR, hazard ratio; ICU, intensive care unit; IQR, interquartile range; MV, mechanical ventilation; NR, not reported; NS, not statistically significant; OCS, oral corticosteroids; OR, odds ratio; PEF, peak expiratory flow; ppb, parts per billion; SARS-CoV-2, severe acute respiratory syndrome coronavirus 2

Changes in asthma management, including treatment escalation or reduced adherence, were more frequently observed following SARS-CoV-2 infection, particularly in studies reporting worsened asthma control [22, 24, 30]. However, longitudinal registry-based studies with extended follow-up generally demonstrated recovery of lung function and symptom control over time, with no consistent evidence of sustained post-acute impairment attributable to SARS-CoV-2 infection alone [28, 29, 31].

### Risk of bias across studies

Risk of bias assessments are presented in Supplementary Table S3. Overall, the methodological quality of included studies ranged from moderate to low risk of bias. Common limitations included reliance on retrospective designs [22–24, 26], unadjusted or partially adjusted analyses [22–24, 30], relatively short post-acute follow-up durations [25, 30], and heterogeneity in outcome definitions across studies.

In contrast, large population-based and registry-based studies with validated exposure ascertainment, clearly defined comparators, and multivariable adjustment, particularly those using national claims or electronic health record databases, generally demonstrated lower risk of bias [27–29, 32].

## Discussion

In this systematic review of 11 observational studies, we synthesised the available evidence on asthma control and exacerbation risk following SARS-CoV-2 infection during the post-acute COVID-19 phase. Overall, the findings indicate heterogeneous post-acute asthma outcomes, with evidence of transient worsening of asthma control and increased exacerbation risk in some populations, particularly following more severe acute COVID-19, but no consistent evidence of sustained or progressive deterioration in asthma control across longer follow-up periods. Pediatric and adult populations demonstrated broadly similar patterns, although the magnitude and persistence of post-acute effects varied substantially by study design, outcome definition, follow-up duration, and comparator framework

The findings of this review extend and refine the existing literature on asthma and COVID-19, which has largely focused on acute infection outcomes such as COVID-19 severity, hospitalisation, and mortality among individuals with asthma [3, 13]. Many prior studies have treated asthma primarily as a baseline comorbidity or risk factor rather than as a condition with its own post-infection disease trajectory [3, 13, 33]. In contrast, the present review specifically evaluated asthma outcomes following recovery from SARS-CoV-2 infection, anchoring analyses to the post-acute phase and requiring asthma-specific control or exacerbation endpoints. Several included studies reported short-term post-acute worsening in asthma control, particularly within the first one to three months after infection, including declines in validated asthma control scores and increased need for treatment escalation [25, 30]. However, these findings were not universal. Larger registry-based and longitudinal studies employing within-person pre–post designs or longer follow-up generally demonstrated stability or recovery of asthma control over time, suggesting that early post-acute deterioration may be temporary rather than sustained [28, 29]

With respect to asthma exacerbations, population-based studies using electronic health records or claims data consistently demonstrated higher post-acute exacerbation risk among individuals who experienced severe or hospitalised COVID-19 [27, 32], while findings were more variable among those with mild or non-hospitalised infection [27, 30, 32]. This apparent gradient by acute COVID-19 severity aligns with broader post-COVID outcome literature and supports a model in which systemic illness severity, rather than infection alone, contributes to downstream asthma morbidity [9]. Importantly, studies evaluating mild or moderate COVID-19 often reported no statistically significant increase in post-acute exacerbations, particularly when outcomes were assessed over shorter follow-up intervals or when analyses relied on unadjusted models [26, 30]

Several mechanisms may plausibly explain the observed post-acute asthma outcomes. Acute SARS-CoV-2 infection has been associated with airway inflammation, immune dysregulation, and epithelial injury, which may transiently worsen asthma control or increase susceptibility to exacerbations [9, 34]. Systemic inflammation, corticosteroid exposure during acute illness, and viral-induced changes in airway responsiveness may also contribute, particularly among patients with severe COVID-19 [9, 34, 35]. In addition, behavioural and healthcare-related factors are likely relevant. Changes in medication adherence, treatment step-down or escalation, healthcare utilisation, and follow-up patterns following COVID-19 may influence observed outcomes, especially in pediatric populations where parental management decisions play a significant role [14, 36]. Notably, several longitudinal studies demonstrated partial or complete recovery of asthma control over time, suggesting that post-acute effects may reflect reversible perturbations rather than permanent alterations in asthma disease course [28, 29]

From a clinical perspective, the available evidence suggests that many individuals may not experience sustained worsening of asthma control following SARS-CoV-2 infection, particularly after mild or moderate disease [28, 29]. However, patients recovering from severe COVID-19 appear to represent a higher-risk subgroup for post-acute asthma exacerbations and may benefit from closer monitoring, proactive follow-up, and reassessment of asthma management in the months following recovery [27, 32]. These results may help reassure clinicians and patients that post-COVID asthma deterioration is not inevitable, while also highlighting the importance of individualised post-acute care, especially among those with severe infection, prior poor asthma control, or significant comorbidities [30, 32]. At a population level, the findings support integration of asthma-specific considerations into post-COVID care pathways without implying a universal long-term asthma burden attributable to SARS-CoV-2 infection [9]. The available evidence does not consistently support routine escalation of asthma therapy following mild COVID-19; however, clinical decisions should remain individualised

### Strengths and limitations

This review has several strengths, including a pre-registered protocol, comprehensive multi-database search strategy, explicit post-acute outcome definitions, and strict inclusion criteria requiring asthma-specific outcomes and appropriate comparators. The focus on validated asthma control measures and clinically relevant exacerbation endpoints enhances interpretability. Nevertheless, important limitations should be acknowledged. The included studies were observational and varied widely in design, outcome definitions, and analytic approaches, precluding quantitative meta-analysis. Residual confounding, misclassification of infection severity or asthma outcomes, and reliance on administrative data in some studies may have influenced results. Follow-up durations were heterogeneous, and few studies provided detailed phenotypic or biomarker data to support mechanistic inference. Evidence also remains limited for certain subgroups, including individuals with severe asthma receiving biologic therapy and those experiencing repeated SARS-CoV-2 infections

Future research should prioritise prospective, longitudinal designs with clearly defined post-acute follow-up intervals, standardised asthma outcome measures, and robust comparator frameworks. Greater attention to COVID-19 severity, reinfection, vaccination status, asthma phenotype, and treatment intensity will be essential to refine risk stratification and guide post-COVID asthma management. Harmonisation of outcome definitions and reporting standards would facilitate meta-analytic synthesis and improve comparability across studies

## Conclusion

In summary, available evidence suggests that asthma control and exacerbation risk following SARS-CoV-2 infection in the post-acute phase are heterogeneous and appear to vary according to infection severity, follow-up duration, and study design. While transient post-acute worsening may occur, particularly after severe COVID-19, the current evidence does not consistently demonstrate sustained deterioration in asthma control across longer-term follow-up. These findings suggest that consideration of infection severity may be relevant in post-COVID asthma management, while underscoring the need for high-quality longitudinal research to better define long-term outcomes

## Supplementary Information

Below is the link to the electronic supplementary material.

Supplementary Material 1.

Supplementary Material 2.

Supplementary Material 3.

## Data Availability

All data generated or analyzed during this study are included in this published article.

## Abbreviations

ACQ: Asthma control questionnaire
ACT: Asthma control test
ACTS: Transformed ACT score and cACT score (collectively)
cACT: Childhood asthma control test
CI: Confidence interval
COPD: Chronic obstructive pulmonary disease
COVID-19: Coronavirus disease 2019
FeNO: Fractional exhaled nitric oxide
FEV₁: Forced expiratory volume in one second
FVC: Forced vital capacity
GINA: Global Initiative for Asthma
HR: Hazard ratio
ICU: Intensive care unit
IQR: Interquartile range
MV: Mechanical ventilation
OCS: Oral corticosteroids
OR: Odds ratio
PCR: Polymerase chain reaction
RAT: Rapid antigen test
RR: Risk ratio
RT-PCR: Reverse transcription polymerase chain reaction
SARS-CoV-2: Severe acute respiratory syndrome coronavirus 2
