# Supplementary material for: Asthma control and exacerbation risk following SARS-CoV-2 infection in the post-acute COVID-19 phase: a systematic review
Source: Allergy Asthma Clin Immunol. 2026 Apr 15;22:36. doi: 10.1186/s13223-026-01027-z (PMC13220527; doi:10.1186/s13223-026-01027-z)
Supplement: Supplementary file 1 — Supplementary Material 1. [file 13223_2026_1027_MOESM1_ESM.docx]

Supplementary Table S1. Database-specific search syntax.

| **Scopus** |
| --- |
| S5 ( TITLE-ABS-KEY ( ( asthma* ) ) ) AND ( TITLE-ABS-KEY ( ( covid* OR "SARS-CoV-2" OR "2019-nCoV" ) ) ) AND ( TITLE-ABS-KEY ( ( "long covid" OR "post-acute" OR "post acute" OR "post covid" OR "long-term" OR "long term" OR persistent OR follow-up ) ) ) AND ( LIMIT-TO ( DOCTYPE , "ar" ) ) AND ( LIMIT-TO ( EXACTKEYWORD , "Human" ) ) AND ( LIMIT-TO ( LANGUAGE , "English" ) ) (1,472)  S4 ( TITLE-ABS-KEY ( ( asthma* ) ) ) AND ( TITLE-ABS-KEY ( ( covid* OR "SARS-CoV-2" OR "2019-nCoV" ) ) ) AND ( TITLE-ABS-KEY ( ( "long covid" OR "post-acute" OR "post acute" OR "post covid" OR "long-term" OR "long term" OR persistent OR follow-up ) ) ) (1,936)  S3 TITLE-ABS-KEY ( ( "long covid" OR "post-acute" OR "post acute" OR "post covid" OR "long-term" OR "long term" OR persistent OR follow-up ) ) (4,986,309)  S2 TITLE-ABS-KEY ( ( covid* OR "SARS-CoV-2" OR "2019-nCoV" ) ) (779,885)  S1 TITLE-ABS-KEY ( ( asthma* ) ) (337,234)  Filters applied:  • Language: English  • Document type: Article  • Source type: Journal |
| **Embase <1974 to 2026 January 21>** |
| 1 exp asthma/ 346062  2 asthma*.ti,ab. 297071  3 1 or 2 387041  4 exp coronavirus disease 2019/ 502132  5 exp severe acute respiratory syndrome coronavirus 2/ 148703  6 covid*.ti,ab. 512790  7 SARS-CoV-2.ti,ab. 169205  8 2019-nCoV.ti,ab. 2097  9 4 or 5 or 6 or 7 or 8 622223  10 exp post-acute covid-19 syndrome/ 12821  11 long covid.ti,ab. 9680  12 post-acute.ti,ab. 12382  13 post acute.ti,ab. 12382  14 post covid.ti,ab. 20234  15 long-term.ti,ab. 1664083  16 long term.ti,ab. 1664083  17 persistent.ti,ab. 485327  18 follow-up.ti,ab. 2366605  19 10 or 11 or 12 or 13 or 14 or 15 or 16 or 17 or 18 4004831  20 3 and 9 and 19 2339  21 limit 21 to (human and english language and journal) 2135 |
| **CINHAL** |
| S4 S3 AND S2 AND S1 (105)  S3 (MH "Post-Acute COVID-19 Syndrome+") OR TI "long covid" OR AB "long covid" OR TI "post-acute" OR AB "post-acute" OR TI "post acute" OR AB "post acute" OR TI "post covid" OR AB "post covid" OR TI "long-term" OR AB "long-term" OR TI "long term" OR AB "long term" OR TI persistent OR AB persistent OR TI "follow-up" OR AB "follow-up" (583,818)  S2 (MH "COVID-19+") OR (MH "SARS-CoV-2+") OR TI covid* OR AB covid* OR TI "SARS-CoV-2" OR AB "SARS-CoV-2" OR TI "2019-nCoV" OR AB "2019-nCoV" (164,100)  S1 (MH "Asthma+") OR TI asthma* OR AB asthma* (53,006)  Limiters applied:  • Language: English  • Peer Reviewed  • Publication Type: Journal Article  • Population: Human |
| **PubMed** |
| **6 Search:** (("Asthma"[MeSH Terms] OR "asthma*"[Title/Abstract]) AND ("COVID-19"[MeSH Terms] OR "SARS-CoV-2"[MeSH Terms] OR "covid*"[Title/Abstract] OR "SARS-CoV-2"[Title/Abstract] OR "2019-nCoV"[Title/Abstract]) AND ("Post-Acute COVID-19 Syndrome"[MeSH Terms] OR "Long COVID"[Title/Abstract] OR "post-acute"[Title/Abstract] OR "post-acute"[Title/Abstract] OR "long-term"[Title/Abstract] OR "long-term"[Title/Abstract] OR "persistent"[Title/Abstract] OR "follow-up"[Title/Abstract])) AND ((humans[Filter]) AND (english[Filter])) (345)  **5 Search:** (("Asthma"[MeSH Terms] OR "asthma*"[Title/Abstract]) AND ("COVID-19"[MeSH Terms] OR "SARS-CoV-2"[MeSH Terms] OR "covid*"[Title/Abstract] OR "SARS-CoV-2"[Title/Abstract] OR "2019-nCoV"[Title/Abstract]) AND ("Post-Acute COVID-19 Syndrome"[MeSH Terms] OR "Long COVID"[Title/Abstract] OR "post-acute"[Title/Abstract] OR "post-acute"[Title/Abstract] OR "long-term"[Title/Abstract] OR "long-term"[Title/Abstract] OR "persistent"[Title/Abstract] OR "follow-up"[Title/Abstract])) AND (humans[Filter]) (363)  **4 Search:** ((( "Asthma"[Mesh] OR asthma*[tiab] )) AND (( "COVID-19"[Mesh] OR "SARS-CoV-2"[Mesh] OR covid*[tiab] OR "SARS-CoV-2"[tiab] OR "2019-nCoV"[tiab] ))) AND (( "Post-Acute COVID-19 Syndrome"[Mesh] OR "Long COVID"[tiab] OR "post-acute"[tiab] OR "post acute"[tiab] OR "long-term"[tiab] OR "long term"[tiab] OR "persistent"[tiab] OR "follow-up"[tiab] )) (525)  **3 Search:** ( "Post-Acute COVID-19 Syndrome"[Mesh] OR "Long COVID"[tiab] OR "post-acute"[tiab] OR "post acute"[tiab] OR "long-term"[tiab] OR "long term"[tiab] OR "persistent"[tiab] OR "follow-up"[tiab] ) (2,621,048)  **2 Search:** ( "COVID-19"[Mesh] OR "SARS-CoV-2"[Mesh] OR covid*[tiab] OR "SARS-CoV-2"[tiab] OR "2019-nCoV"[tiab] ) (492,192)  **1 Search:** ( "Asthma"[Mesh] OR asthma*[tiab] ) (220,483) |
| **Web of Science Core Collection** |
| S5 asthma* (Topic) and covid* OR "SARS-CoV-2" OR "2019-nCoV" (Topic) and "long covid" OR "post-acute" OR "post acute" OR "post covid" OR "long-term" OR "long term" OR persistent OR follow-up (Topic) and Article (Document Types) and English (Languages) and Science Citation Index Expanded (SCI-EXPANDED) or Social Sciences Citation Index (SSCI) (Web of Science Index) and Article (Document Types) and English (Languages) (447)  S4 asthma* (Topic) and covid* OR "SARS-CoV-2" OR "2019-nCoV" (Topic) and "long covid" OR "post-acute" OR "post acute" OR "post covid" OR "long-term" OR "long term" OR persistent OR follow-up (Topic) (738)  S3 "long covid" OR "post-acute" OR "post acute" OR "post covid" OR "long-term" OR "long term" OR persistent OR follow-up (Topic) (3,750,016)  S2 covid* OR "SARS-CoV-2" OR "2019-nCoV" (Topic) (672,029)  S1 asthma* (Topic) (273,288)  Filters applied:  • Document type: Article  • Language: English  • Web of Science Indexes: Science Citation Index Expanded (SCI-EXPANDED) and Social Sciences Citation Index (SSCI) |
