# Supplementary material for: Asthma control and exacerbation risk following SARS-CoV-2 infection in the post-acute COVID-19 phase: a systematic review
Source: Allergy Asthma Clin Immunol. 2026 Apr 15;22:36. doi: 10.1186/s13223-026-01027-z (PMC13220527; doi:10.1186/s13223-026-01027-z)
Supplement: Supplementary file 2 — Supplementary Material 2. [file 13223_2026_1027_MOESM2_ESM.docx]

Supplementary Table S2. Studies excluded at full-text stage.

| Article excluded | Reason for exclusion |
| --- | --- |
| Afsin E, Demirkol ME. Post-COVID pulmonary function test evaluation. Turkish Thoracic Journal. 2022;23(6):387–394. | Evaluated post-COVID pulmonary function and functional status (spirometry, hypoxemia, Post-COVID-19 Functional Status Scale) in a mixed population, without asthma-specific analyses or reporting of asthma control (ACT, cACT, ACQ, or GINA control) or asthma exacerbation outcomes. Patients with pre-existing lung disease (asthma, COPD, ILD) were not analysed separately, and no uninfected asthma comparator or asthma-anchored pre–post analysis was provided. |
| Alsayed AR, Talib W, Al-Dulaimi A, Daoud S, Al Maqbali M. The first detection of Pneumocystis jirovecii in asthmatic patients post-COVID-19 in Jordan. Bosnian Journal of Basic Medical Sciences. 2022;22(5):784–790. | Evaluated Pneumocystis jirovecii detection and coinfection in asthmatic patients following SARS-CoV-2 infection, with outcomes focused on microbiological detection and coinfection prevalence. The study did not report asthma-specific post-acute outcomes, such as asthma control (ACT, cACT, ACQ, or GINA control) or asthma exacerbation risk, and lacked an uninfected asthma comparator or asthma-anchored pre–post analysis relevant to post-acute (≥4 weeks) asthma outcomes. |
| Amat F, Delaisi B, Labbé J-P, Leonardi J, Houdouin V. Asthma may not be a risk factor for severe COVID-19 in children. Journal of Allergy and Clinical Immunology: In Practice. 2021;9(6):2478–2479. | Single-centre pediatric cluster study evaluating acute and short-term outcomes of SARS-CoV-2 infection in children with asthma, with follow-up limited to ~1 month after infection. The study focused on COVID-19 severity and short-term asthma status, did not assess post-acute (≥4 weeks) asthma control or exacerbation risk using validated longitudinal outcome measures, and lacked an uninfected asthma comparator or asthma-anchored pre–post analysis relevant to post-acute outcomes. |
| Antonogiannaki E-M, Grigoropoulos I, Manali ED, Thomas K, Kallieri M, Alexopoulou P, et al. Long-Term Lung Sequelae in Survivors of Severe/Critical COVID-19 Pneumonia: The “Non-Steroid”, “Non-Interventional” Approach. Journal of Clinical Medicine. 2025;14:347. | Prospective follow-up study of survivors of severe/critical COVID-19 pneumonia evaluating long-term pulmonary imaging (HRCT), lung function (FEV₁, FVC, DLCO), dyspnea (mMRC), and quality-of-life (SGRQ) outcomes in a general post-COVID population. Asthma was reported only as a minor comorbidity and not analysed separately, and asthma-specific post-acute outcomes (asthma control or asthma exacerbation risk) were not assessed, nor were asthma-anchored comparators used. |
| Avdeev SN, Gaynitdinova VV, Chikina SY, Merzhoeva ZM, Nuralieva GS, Gneusheva TY, et al. Association between eosinophil counts and outcomes of severe coronavirus disease 2019 in elderly asthma patients: a prospective cohort study. Journal of Thoracic Disease. 2025;17(12):11283–11293. | Prospective cohort study evaluating in-hospital and early (90-day) post-discharge mortality and predictors of death (eosinophil count, comorbidity index, CT severity) in elderly patients with asthma and severe COVID-19. The study focused on mortality outcomes and prognostic biomarkers, and did not assess asthma-specific post-acute outcomes, such as asthma control (ACT, cACT, ACQ, or GINA control) or asthma exacerbation risk, nor did it evaluate the post-infection asthma disease course. |
| Avdeev SN, Gaynitdinova VV, Pozdnakova AA, Chikina SY, Kosobokova KA. Asthma in elderly and coronavirus disease 2019: Results of 90-day post-hospital follow-up. Annals of Allergy, Asthma & Immunology. 2023;131(5):663–664. | Evaluated short-term (90-day) post-hospital mortality and predictors of death among elderly patients with asthma following severe COVID-19. The study focused on survival outcomes and prognostic factors (e.g., comorbidity burden, CT lesion extent, eosinophil count) rather than post-acute (≥4 weeks) asthma-specific outcomes. It did not assess asthma control using validated instruments (ACT, cACT, ACQ, or GINA control), did not evaluate asthma exacerbation risk (e.g., systemic corticosteroid use, emergency department visits, or asthma-related hospitalisation), and lacked an appropriate asthma-specific comparator or longitudinal pre–post assessment of asthma outcomes. |
| Ayache M, Khalid F. Increased likelihood of COVID-19-related asthma exacerbation with higher eosinophil count. Ann Allergy Asthma Immunol. 2025;135(3):328–329. | Retrospective cross-sectional study evaluating asthma exacerbations occurring within 4 weeks of confirmed SARS-CoV-2 infection, focusing on acute or peri-acute COVID-19–related asthma exacerbations and phenotypic risk modifiers (blood eosinophil count, BMI), rather than assessing post-acute (≥4 weeks) asthma control or exacerbation outcomes following recovery from infection. No longitudinal post-acute follow-up or asthma control assessment was performed. |
| Basin S, Valentin S, Maurac A, Poussel M, Pequignot B, Brindel A, et al. Progression to a severe form of COVID-19 among patients with chronic respiratory diseases. Respiratory Medicine and Research. 2022;81:100880. | Multicentre retrospective study evaluating progression to severe acute COVID-19 (WHO Clinical Progression Scale ≥6), ICU admission, and in-hospital outcomes among patients with chronic respiratory diseases, including asthma and COPD. The study focused on acute COVID-19 severity during hospitalization, analysed asthma within heterogeneous chronic respiratory disease groups, and did not assess asthma-specific post-acute outcomes, such as asthma control or asthma exacerbation risk following SARS-CoV-2 infection (≥4 weeks). |
| Beurnier A, Jutant E-M, Jevnikar M, Boucly A, Pichon J, Preda M, et al. Characteristics and outcomes of asthmatic patients with COVID-19 pneumonia who require hospitalisation. European Respiratory Journal. 2020;56:2001875. | Prospective monocentric cohort evaluating acute COVID-19 pneumonia severity, ICU admission, and short-term (≈1-month) outcomes in hospitalised patients with asthma. The study focused on acute disease characteristics and outcomes, compared asthmatic versus non-asthmatic COVID-19 pneumonia patients, and did not assess asthma-specific post-acute outcomes, such as asthma control (ACT, cACT, ACQ, or GINA control) or asthma exacerbation risk following SARS-CoV-2 infection (≥4 weeks). |
| Beurnier A, Yordanov Y, Dechartres A, Dinh A, Debuc E, Lescure F-X, et al. Characteristics and outcomes of asthmatic outpatients with COVID-19 who receive home telesurveillance. ERJ Open Research. 2022;8:00012-2022. | Large prospective cohort of asthmatic outpatients with suspected or confirmed COVID-19 managed through home telesurveillance, evaluating clinical worsening (hospitalisation or death within 30 days) and COVID-19–related care escalation. The study did not assess asthma-specific post-acute outcomes, such as asthma control (ACT, cACT, ACQ, or GINA control) or asthma exacerbation risk, and outcomes were focused on COVID-19 severity and healthcare utilisation rather than the post-infection asthma disease course. |
| Bloom CI, Drake TM, Docherty AB, Lipworth BJ, Johnston SL, Nguyen-Van-Tam JS, et al. Risk of adverse outcomes in patients with underlying respiratory conditions admitted to hospital with COVID-19: a national, multicentre prospective cohort study using the ISARIC WHO Clinical Characterisation Protocol UK. The Lancet Respiratory Medicine. 2021;9:699–711. | Prospective multicentre cohort study evaluating acute in-hospital COVID-19 outcomes, including mortality, critical care admission, and ventilatory support, in patients with asthma and other chronic respiratory conditions. Asthma was analysed as a baseline comorbidity influencing acute COVID-19 severity, and the study did not assess post-acute (≥4 weeks) asthma-specific outcomes, such as asthma control or post-infection exacerbation risk following recovery from SARS-CoV-2 infection, which were the primary outcomes of interest for this review. |
| Bonifazi M, Mei F, Skrami E, Latini LL, Amico D, Balestro E, et al. Predictors of Worse Prognosis in Young and Middle-Aged Adults Hospitalized with COVID-19 Pneumonia: A Multi-Center Italian Study (COVID-UNDER50). Journal of Clinical Medicine. 2021;10:1218. | Multi-centre retrospective study evaluating acute COVID-19 prognosis (need for non-invasive/invasive mechanical ventilation and in-hospital mortality) in young and middle-aged adults hospitalised with COVID-19 pneumonia. Asthma was analysed only as a comorbidity/predictor of acute outcomes, without assessment of asthma-specific post-acute outcomes, such as asthma control (ACT, cACT, ACQ, or GINA control) or asthma exacerbation risk following SARS-CoV-2 infection (≥4 weeks). |
| Campos CL, Nguyen C, Crothers K, Awan O, Miller FJ Jr, Sedillo C, et al. Post–COVID-19 syndrome clinical pathway for the US Veterans Health Administration. Journal of Clinical Pathways. 2023;9(1):22–28. | Consensus-based clinical pathway and guidance document describing diagnostic evaluation and management of post–COVID-19 dyspnoea and cough within the US Veterans Health Administration. The article does not present original patient-level data or comparative analyses and does not evaluate asthma-specific post-acute outcomes (e.g., asthma control or asthma exacerbation risk) following SARS-CoV-2 infection, which were the primary outcomes of interest for this review. |
| Chiu MN, Chowdhury A, Zhang K, et al. Respiratory burden in post-acute COVID-19 sequelae: a longitudinal study of airway and systemic inflammation and clinical outcomes. ERJ Open Research. 2025. | Study population explicitly excluded individuals with pre-existing chronic respiratory diseases, including asthma. The study focused on respiratory symptom burden and inflammatory biomarkers in previously healthy adults with post-acute COVID-19 sequelae (PACS), without assessment of post-acute asthma control using validated asthma-specific instruments (ACT, cACT, or ACQ) or evaluation of asthma exacerbation outcomes. |
| Deivendran G, Kanagaraj TS, Leelabai BS, et al. Heart rate variability in young adults with bronchial asthma with and without previous COVID-19 infection: A cross-sectional study. Journal of Clinical and Diagnostic Research. 2025;19(8):CC01–CC04. | Cross-sectional study focusing on autonomic nervous system function assessed by heart rate variability (HRV) and haemodynamic parameters. The study did not evaluate post-acute asthma outcomes, did not assess asthma control using validated instruments (ACT, cACT, or ACQ), and did not examine asthma exacerbation risk or longitudinal post-SARS-CoV-2 asthma trajectories. |
| DeVries A, Shambhu S, Sloop S, Overhage JM. One-year adverse outcomes among US adults with post–COVID-19 condition vs those without COVID-19 in a large commercial insurance database. JAMA Health Forum. 2023;4(3):e230010. | Large population-based cohort study of adults with post–COVID-19 condition (PCC) assessing incident diagnoses and adverse outcomes using administrative claims data. Asthma was analysed as a baseline comorbidity or incident claims-based diagnosis, rather than as a predefined asthma cohort. The study did not evaluate post-acute asthma control using validated instruments (ACT, cACT, or ACQ) and did not assess asthma-specific exacerbation outcomes following SARS-CoV-2 infection. |
| Di Chiara C, Carraro S, Zanconato S, et al. Preliminary evidence on pulmonary function after asymptomatic and mild COVID-19 in children. Children. 2022;9:952. | The study population excluded children with pre-existing chronic respiratory diseases, including asthma, and therefore did not evaluate post-acute outcomes in an asthma cohort. Outcomes were limited to spirometric lung function in generally healthy children following asymptomatic or mild SARS-CoV-2 infection, without assessment of asthma control using validated instruments (ACT, cACT, or ACQ) or asthma exacerbation risk. |
| Duong KS, Henry SS, Duong TQ. Severe acute respiratory syndrome coronavirus 2 infection and the long-term risk of pneumonia in an urban population: An observational cohort study up to 46 months after infection. Clinical Infectious Diseases. 2025; c iaf345. | Population-based cohort study evaluating new-onset pneumonia following SARS-CoV-2 infection in the general population. Asthma was included only as a baseline comorbidity/covariate, rather than as a predefined asthma cohort. The study did not assess post-acute asthma control using validated instruments (ACT, cACT, or ACQ) and did not evaluate asthma-specific exacerbation outcomes (e.g., systemic corticosteroid use, asthma-related emergency department visits, or hospitalisation). |
| Eggert LE, He Z, Collins W, et al. Asthma phenotypes, associated comorbidities, and long-term symptoms in COVID-19. Allergy. 2022;77:173–185. | The study primarily examined asthma phenotypes, hospitalization risk, COVID-19 disease severity, and general long-term COVID-19 symptoms. It did not evaluate post-acute asthma outcomes, including asthma control using validated instruments (ACT, cACT, or ACQ) or asthma-specific exacerbation outcomes (e.g., systemic corticosteroid use, asthma-related emergency department visits, or hospitalisation) following SARS-CoV-2 infection. |
| Eguiluz-Gracia I, van den Berge M, Boccabella C, et al. Real-life impact of COVID-19 pandemic lockdown on the management of pediatric and adult asthma: A survey by the EAACI Asthma Section. Allergy. 2021;76:2776–2784. | Multinational survey of healthcare professionals evaluating asthma management practices and healthcare delivery during COVID-19 lockdown. The study did not include patient-level data on SARS-CoV-2 infection and did not assess post-acute asthma outcomes, including asthma control using validated instruments (ACT, cACT, or ACQ) or asthma-specific exacerbation outcomes following infection. |
| Ekström S, Mogensen I, Georgelis A, et al. General stress among young adults with asthma during the COVID-19 pandemic. Journal of Allergy and Clinical Immunology: In Practice. 2022;10:108–115. | Population-based cross-sectional study examining COVID-19–related anxiety, health concerns, and perceived stress among young adults with asthma. The study did not assess post-acute asthma outcomes, including asthma control using validated instruments (ACT, cACT, or ACQ) or asthma-specific exacerbation outcomes (e.g., systemic corticosteroid use, asthma-related emergency department visits, or hospitalisation) following SARS-CoV-2 infection. |
| Elneima O, Hurst JR, Echevarria C, et al. Long-term impact of COVID-19 hospitalisation among individuals with pre-existing airway diseases in the UK: a multicentre, longitudinal cohort study (PHOSP-COVID). ERJ Open Research. 2024;10:00982-2023. | Multicentre cohort of hospitalised adults with mixed pre-existing airway diseases (asthma, COPD, bronchiectasis) analysed largely as a combined airways group. Outcomes focused on post-COVID recovery, symptom burden, physical performance, mental health and health-related quality of life, without assessment of post-acute asthma control using validated instruments (ACT, cACT, or ACQ) or asthma-specific exacerbation outcomes (e.g., systemic corticosteroid use, asthma-related emergency department visits, or hospitalisation). |
| Esmaeilzadeh H, Sanaei Dashti A, Mortazavi N, et al. Persistent cough and asthma-like symptoms post COVID-19 hospitalization in children. BMC Infectious Diseases. 2022;22:244. | Prospective paediatric cohort of hospitalised COVID-19 patients evaluating new-onset “asthma-like” symptoms and persistent cough after discharge, rather than post-acute outcomes in a predefined asthma population. The study did not assess asthma control using validated instruments (ACT, cACT, or ACQ) and did not evaluate asthma-specific exacerbation outcomes with an appropriate comparator. |
| Fan H, Ren Z, Zhang P, Zhou B. Increased asthma burden during and after the COVID-19 pandemic among US adults. Ann Allergy Asthma Immunol. 2025;134(6):731–738. | Population-level cross-sectional analysis evaluating changes in asthma prevalence and burden across pre-pandemic and pandemic/post-pandemic periods using NHANES survey data, without individual-level classification by confirmed SARS-CoV-2 infection or assessment of post-acute (≥4 weeks) asthma control or exacerbation outcomes following infection. Asthma was analysed as a population outcome rather than as a predefined cohort with infection-anchored comparators. |
| Farrokhpour M, Safarnezhad Tameshkel F, Kalaki NS, et al. Association of inflammatory scoring tools with spirometry indices in COVID-19 patients: a single-center cohort study. Iranian Journal of Microbiology. 2025;17(6):1042–1048. | Single-center cohort of general COVID-19 patients evaluating associations between inflammatory biomarkers (e.g., CRP, NLR, MPV, APACHE II) and spirometry indices at 3-month follow-up. Asthma was included only as a baseline comorbidity, and the study did not assess post-acute asthma control using validated instruments (ACT, cACT, or ACQ) or asthma-specific exacerbation outcomes in a predefined asthma population. |
| Farzan S, Rai S, Cerise J, et al. Asthma and COVID-19: An early inpatient and outpatient experience at a US children’s hospital. Pediatric Pulmonology. 2021;56:2522–2529. | Single-center retrospective study primarily evaluating acute COVID-19 severity, inpatient management, and hospital outcomes in children with and without asthma, as well as outpatient asthma management behaviors during the pandemic. The study did not assess post-acute (≥4 weeks) asthma outcomes, did not evaluate asthma control using validated instruments (ACT, cACT, or ACQ), and did not examine post-infection asthma exacerbation risk following SARS-CoV-2 infection. |
| Faverio P, Luppi F, Rebora P, et al. Six-month pulmonary impairment after severe COVID-19: A prospective, multicentre follow-up study. Respiration. 2021;100:107–118. | Prospective multicentre cohort of adults hospitalised for severe COVID-19 pneumonia evaluating pulmonary sequelae (DLCO, spirometry, radiology, 6-MWT, dyspnoea) at 6 months. Asthma was included only as a baseline comorbidity, and the study did not assess post-acute asthma control using validated instruments (ACT, cACT, or ACQ) or asthma-specific exacerbation outcomes following SARS-CoV-2 infection. |
| Fernández-de-las-Peñas C, Torres-Macho J, Velasco-Arribas M, et al. Similar prevalence of long-term post-COVID symptoms in patients with asthma: A case-control study. Journal of Infection. 2021;83:237–279. | Case–control study evaluating long-term post-COVID (long-COVID) symptom prevalence (e.g., fatigue, dyspnoea, cognitive symptoms, sleep quality, anxiety/depression) in patients with and without asthma. The study did not assess post-acute asthma-specific outcomes, including asthma control using validated instruments (ACT, cACT, or ACQ) or asthma exacerbation outcomes (e.g., systemic corticosteroid use, asthma-related emergency department visits, or hospitalisation) following SARS-CoV-2 infection. |
| Finkas LK, Ramesh N, Block LS, et al. Asthma and COVID-19 outcomes: A prospective study in a large health care delivery system. Journal of Asthma and Allergy. 2023;16:1041–1051. | Large prospective cohort study evaluating acute COVID-19 outcomes (SARS-CoV-2 infection, COVID-19–related hospitalisation, ICU admission, and mortality) in patients with asthma compared with matched controls across different pandemic periods. The study did not assess post-acute asthma outcomes, including asthma control using validated instruments (ACT, cACT, or ACQ), nor did it evaluate post-infection asthma exacerbation risk following SARS-CoV-2 infection. |
| Hamadneh M, Alquran A, Manna R. Impact of the COVID-19 on asthma control among children: A systematic review. Journal of Public Health Research. 2023;12:22799036231197186. | Systematic review evaluating the impact of COVID-19 lockdowns and pandemic-related public health measures on pediatric asthma outcomes (asthma control, exacerbations, emergency department visits, hospitalisations, and psychosocial effects). The study did not assess asthma outcomes following confirmed individual-level SARS-CoV-2 infection, and focused on pandemic-period and lockdown effects rather than post-acute (≥4 weeks) asthma outcomes after COVID-19, which were the primary outcomes of interest for this review. |
| Hasan SS, Capstick T, Razi Zaidi ST, Kow CS, Merchant HA. Use of corticosteroids in asthma and COPD patients with or without COVID-19. Respiratory Medicine. 2020;170:106045. | Narrative review article discussing the safety and clinical use of inhaled and oral corticosteroids in asthma and COPD patients during the COVID-19 pandemic. The article did not report original patient-level data and did not assess post-acute asthma outcomes, including asthma control using validated instruments (ACT, cACT, or ACQ) or asthma-specific exacerbation outcomes following SARS-CoV-2 infection. |
| Hernandez Santiago V, Fagbamigbe AF, Sullivan FM, Agrawal U, Morales D, McCowan C, Lipworth B. Intranasal steroid use and COVID-19 mortality among patients with asthma and COPD: A retrospective cohort study. Annals of Allergy, Asthma & Immunology. 2023;131:474–481. | Retrospective population-based cohort study evaluating the association between intranasal corticosteroid (INCS) exposure and COVID-19–related and all-cause mortality. Although asthma and COPD subgroups were analysed, the study did not assess post-acute asthma outcomes, including asthma control using validated instruments (ACT, cACT, or ACQ), nor did it evaluate asthma-specific exacerbation outcomes following SARS-CoV-2 infection. |
| Huang C-Y, Wu Y-K, Yang M-C, et al. Assessing post-COVID-19 respiratory dynamics: a comprehensive analysis of pulmonary function, bronchial hyperresponsiveness and bronchodilator response. ERJ Open Research. 2024;10:00149-2024. | Single-centre observational study evaluating airway hyperresponsiveness (methacholine challenge), spirometry changes, and bronchodilator response in adults with post-COVID-19 respiratory symptoms. The study did not include a predefined asthma cohort and focused on post-COVID respiratory physiology and potential new-onset airway hyperreactivity, rather than post-acute asthma outcomes. Asthma control was not assessed using validated instruments (ACT, cACT, or ACQ), and asthma-specific exacerbation outcomes were not evaluated. |
| Hung C-T, Hung Y-C, Suk C-W, Wu C-H. Evaluating the associations among asthma, asthma control and long COVID in U.S. adults. Infection. 2025;53:2523–2531. | Cross-sectional, nationally representative survey study (NHIS) examining long COVID prevalence and severity and its association with asthma and asthma control. The study evaluated asthma as a risk factor for long COVID, rather than assessing post-acute asthma outcomes following SARS-CoV-2 infection. It did not measure asthma control using validated instruments (ACT, cACT, or ACQ) and did not evaluate asthma-specific exacerbation outcomes (e.g., systemic corticosteroid use, asthma-related emergency department visits or hospitalisation) attributable to post-acute COVID-19. |
| Hung C-T, Hung Y-C, Suk C-W. Prevalence and characteristics in long COVID among adults with asthma in the United States. Journal of Asthma. 2024;61(7):736–744. | Cross-sectional, nationally representative survey study (NHIS 2022) evaluating the prevalence and risk factors of long COVID among adults with asthma. The study assessed asthma as a risk factor for long COVID and examined sociodemographic predictors and proxy markers of asthma control (self-reported asthma attacks, ER visits), but did not evaluate post-acute asthma outcomes following SARS-CoV-2 infection. Asthma control was not assessed using validated instruments (ACT, cACT, or ACQ), and asthma-specific exacerbation outcomes attributable to post-acute COVID-19 were not evaluated longitudinally. |
| Jaleel A, Namoos K, Asim S, Uppal SS, Zaman S, Irfan H, et al. Interplay of interleukins (IL-6, IL-10) and 25-hydroxycholecalciferol in asthmatic subjects with chronic post-COVID condition (PCC). Tropical Biomedicine. 2024;41(1):70–77. | Cross-sectional biomarker-focused study evaluating inflammatory cytokines (IL-6, IL-10) and vitamin D status in asthmatic and non-asthmatic individuals with and without post-COVID condition. The study did not assess post-acute asthma outcomes, including asthma control using validated instruments (ACT, cACT, or ACQ), nor did it evaluate asthma-specific exacerbation outcomes (e.g., systemic corticosteroid use, asthma-related emergency department visits, or hospitalisation) following SARS-CoV-2 infection. |
| Karaatmaca B, Selmanoğlu A, Toyran M, Emeksiz ZŞ, Şenses Dinç G, Öden Akman A, et al. Quality of life and the psychological status of the adolescents with asthma and their parents during the COVID-19 pandemic. Turkish Journal of Pediatrics. 2022;64(5):805–815. | Cross-sectional study evaluating quality of life and anxiety among adolescents with asthma and their parents during the COVID-19 pandemic. The study explicitly reported no history of SARS-CoV-2 infection among participants and assessed pandemic-related psychosocial effects rather than post-acute (≥4 weeks) asthma outcomes following confirmed infection. Asthma control (ACT) was evaluated independently of SARS-CoV-2 exposure, and no post-infection asthma control or exacerbation outcomes with appropriate comparators or longitudinal pre–post assessment were reported. |
| Kerget B, Araz Ö, Akgün M. The role of exhaled nitric oxide (FeNO) in the evaluation of lung parenchymal involvement in COVID-19 patients. Internal and Emergency Medicine. 2022;17:1951–1958. | Hospital-based observational study evaluating fractional exhaled nitric oxide (FeNO) as a biomarker of acute and in-hospital COVID-19 severity and lung parenchymal involvement. The study explicitly excluded patients with asthma and other chronic airway diseases, and outcomes were limited to FeNO levels, chest CT scores, and inflammatory markers during hospitalization and at discharge. It did not assess post-acute (≥4 weeks) asthma-specific outcomes, asthma control (ACT, cACT, ACQ), or asthma exacerbation risk, and lacked an asthma-anchored comparator or longitudinal assessment of asthma outcomes following SARS-CoV-2 infection. |
| Kim BG, Lee H, Yeom SW, Jeong CY, Park DW, Park TS, et al. Increased risk of new-onset asthma after COVID-19: A nationwide population-based cohort study. Journal of Allergy and Clinical Immunology: In Practice. 2024;12:120–132. | Nationwide population-based cohort study evaluating the incidence of new-onset asthma following SARS-CoV-2 infection in individuals without pre-existing asthma at baseline. The study did not assess post-acute (≥4 weeks) asthma-specific outcomes—such as asthma control, exacerbation risk, or healthcare utilisation—among patients with established asthma, which were the primary outcomes of interest for this review. |
| Kim SH, Kang MG, Jang JG, et al. Severe COVID-19, vaccination, and mortality among individuals with asthma: A nationwide population-based cohort study. Vaccine. 2026. | The study primarily evaluated all-cause mortality in individuals with asthma in relation to COVID-19 vaccination status and severe COVID-19, rather than post-acute asthma outcomes. It did not assess post-acute asthma control using validated instruments (ACT, cACT, or ACQ) or evaluate asthma exacerbation risk following SARS-CoV-2 infection as a primary outcome. |
| Laorden D, Domínguez-Ortega J, Carpio C, Barranco P, Villamañán E, Romero D, et al. Long COVID outcomes in an asthmatic cohort and its implications for asthma control. Respiratory Medicine. 2023;207:107092. | Descriptive, single-centre retrospective cohort of asthmatic adults hospitalised with COVID-19, primarily evaluating long-COVID symptomatology (e.g., dyspnoea, chest pain, cough) and acute disease severity. Although post-COVID ACT scores and mean exacerbation counts were reported, the study lacked an uninfected asthma comparator and did not perform an analytic asthma-anchored pre–post comparison of post-acute asthma control or exacerbation risk attributable to SARS-CoV-2 infection. Outcomes were reported descriptively without comparative effect estimates (RR/OR/HR) relevant to post-acute (≥4 weeks) asthma disease trajectory. |
| Lara ST, Rein LE, Simanek AM, Totoraitis MF, Rausch DJ, Weston BW, et al. Asthma as a risk factor for hospitalization in children and youth with COVID-19: A retrospective cohort study. Pediatric Infectious Disease Journal. 2024;43(5):437–443. | Retrospective population-based cohort study evaluating asthma as a baseline risk factor for acute COVID-19 hospitalization among children and youth. The study focused on predictors of acute disease severity at the time of SARS-CoV-2 infection and did not assess post-acute (≥4 weeks) asthma-specific outcomes. Asthma control (ACT, cACT, ACQ, or GINA control), asthma exacerbation risk after recovery, and longitudinal post-infection asthma outcomes were not evaluated, and asthma was treated as an exposure rather than as an outcome of interest following SARS-CoV-2 infection. |
| Leo F, Bülau JE, Semper H, Grohé C. Correlation of respiratory muscle function and cardiopulmonary exercise testing in post-acute COVID-19 syndrome. Infection. 2023;51:527–530. | Small case series evaluating respiratory muscle function (maximum inspiratory pressure, mouth occlusion pressure) and cardiopulmonary exercise testing parameters in patients with post-acute COVID-19 syndrome. The study population was not restricted to individuals with pre-existing asthma (only a minority had mild, controlled asthma), and analyses were not asthma-anchored. Outcomes focused on physiological and exercise capacity measures rather than asthma-specific post-acute outcomes. Asthma control (ACT, cACT, ACQ), asthma exacerbation risk, and longitudinal post-infection asthma outcomes were not assessed, and no asthma-specific comparator or pre–post asthma analysis was performed. |
| Matsuyama E, Miyata J, Terai H, et al. Chronic obstructive pulmonary disease, asthma, and mechanical ventilation are risk factors for dyspnea in patients with long COVID: A Japanese nationwide cohort study. Respiratory Investigation. 2024;62:1094–1101. | Nationwide cohort study evaluating persistent dyspnea as a long-COVID symptom among hospitalised COVID-19 survivors, with asthma analysed only as a baseline comorbidity/risk factor. The study did not assess asthma-specific post-acute outcomes, such as asthma control (ACT, cACT, ACQ), asthma exacerbation risk, or medication changes, nor did it include asthma-anchored comparators or pre–post infection analyses within an asthma cohort. |
| Melinte OE, Robu Popa D, Dobrin ME, et al. Assessment of Some Risk Factors and Biological Predictors in the Post COVID-19 Syndrome in Asthmatic Patients. Journal of Personalized Medicine. 2024;14:21. | Small single-centre retrospective cohort (n = 37) focusing on biological and laboratory predictors (e.g., CRP, LDH, ALT/AST, glucose, hematological parameters) in asthmatic patients with post-COVID-19 syndrome. The study did not assess asthma-specific post-acute clinical outcomes, such as asthma control (ACT, cACT, ACQ), asthma exacerbation risk, or asthma medication changes, and lacked asthma-anchored comparators or longitudinal pre–post infection analyses of asthma outcomes. |
| Meng M, Wei R, Wu Y, et al. Long-term risks of respiratory diseases in patients infected with SARS-CoV-2: a longitudinal, population-based cohort study. eClinicalMedicine. 2024;69:102500. | Large population-based cohort study evaluating the incidence of newly diagnosed respiratory diseases, including asthma, following SARS-CoV-2 infection in the general adult population. Asthma was analysed as an incident outcome, rather than as a pre-existing condition, and the study did not assess post-acute asthma-specific outcomes such as asthma control, exacerbation risk, medication changes, or healthcare utilisation among patients with established asthma. |
| Metbulut AP, Yılmaz D, Külhaş Çelik İ, Civelek E, Dibek Mısırlıoğlu E, Toyran M. Long-Term Effects of COVID-19 on Respiratory Symptoms and Asthma Control in Pediatric Patients with Asthma. Turkish Journal of Pediatric Disease. 2024;18:117–123. | Single-centre retrospective paediatric cohort primarily evaluating persistent post-COVID respiratory symptoms (e.g., cough, dyspnoea, chest pain) using telephone-based follow-up. Although asthma control status (GINA categories) and exacerbation frequencies were reported descriptively, the study lacked an uninfected asthma comparator and did not perform an analytic asthma-anchored pre–post comparison with comparative effect estimates (RR/OR/HR) to quantify post-acute (≥4 weeks) changes in asthma control or exacerbation risk attributable to SARS-CoV-2 infection. |
| Muntean IA, Leru PM, Pintea I, Bocsan IC, Dobrican CT, Deleanu D. A retrospective study regarding the influence of COVID-19 disease on asthma. BMC Pulmonary Medicine. 2023;23:22. | Retrospective single-centre cohort evaluating asthma control and symptom worsening predominantly during or immediately following acute COVID-19 illness, with repeated monitoring throughout the pandemic period. Although asthma control (ACT, VAS) and exacerbation-like worsening were described, the study did not clearly define or isolate a post-acute (≥4 weeks) follow-up window, lacked an uninfected asthma comparator, and did not provide analytic post-acute effect estimates (RR/OR/HR) for asthma control or exacerbation risk attributable to SARS-CoV-2 infection. Outcomes were largely descriptive and focused on acute/pandemic-period asthma management rather than post-acute asthma disease trajectory. |
| Nursoy MA, Bülbül L, Yazıcı M, et al. Comparison of clinical features of COVID-19 infection in children with asthma and their healthy peers. Pediatric Pulmonology. 2022;57:1693–1700. | Pediatric case–control study evaluating the acute clinical course of COVID-19 in children with asthma compared with healthy peers, focusing on symptoms, hospitalisation, and asthma exacerbations during the acute infection phase. The study did not assess post-acute (≥4 weeks) asthma outcomes, such as asthma control after recovery, longitudinal asthma trajectory, or post-COVID exacerbation risk, and therefore did not meet the review’s outcome and timing eligibility criteria. |
| Panichaporn W, Boonard K, Kanchanapoomi K, et al. Level of asthma control in children and adolescents before and during the COVID-19 pandemic. Asian Pacific Journal of Allergy and Immunology. 2025;43(2):312–319. | Pandemic-period observational study comparing asthma control, exacerbations, spirometry, and inhaled corticosteroid adherence before versus during the COVID-19 pandemic, with outcomes primarily attributed to lockdown-related behavioural and environmental factors (e.g., mask wearing, reduced viral exposure, lifestyle changes). Asthma outcomes were not analysed as post-acute (≥4 weeks) sequelae following confirmed SARS-CoV-2 infection, and infection status was not used as the primary exposure, thus not meeting the review’s exposure and timing eligibility criteria. |
| Papaioannou AI, Fouka E, Tzanakis N, et al. SARS-CoV-2 infection in severe asthma patients treated with biologics. Journal of Allergy and Clinical Immunology: In Practice. 2022;10:2588–2595. | Prospective multicentre cohort evaluating acute SARS-CoV-2 infection risk, COVID-19 severity, and short-term asthma exacerbations during active infection in patients with severe asthma receiving biologic therapy. The study did not assess post-acute (≥4 weeks) asthma outcomes following recovery, such as asthma control, long-term exacerbation risk, or changes in asthma trajectory, and therefore did not meet the review’s outcome timing eligibility criteria. |
| Philip KEJ, Buttery S, Williams P, et al. Impact of COVID-19 on people with asthma: a mixed methods analysis from a UK-wide survey. BMJ Open Respiratory Research. 2022;9:e001056. | UK-wide survey-based mixed methods study relying on self-reported COVID-19 status and subjective asthma-related outcomes (e.g., perceived worsening of asthma management, increased inhaler use, breathing changes). The study lacked confirmed SARS-CoV-2 infection anchoring, did not apply a defined post-acute (≥4 weeks) follow-up window, and did not assess validated asthma control measures or objective exacerbation outcomes (e.g., ACT/ACQ, systemic corticosteroid use, healthcare utilisation), and therefore did not meet the review’s exposure, timing, and outcome eligibility criteria. |
| Qian J, Zhang G, Wu H, Sha L. Impact of the COVID-19 infection on children with allergic diseases in China. Pediatric Research. 2025;97:360–364. | Retrospective single-centre study comparing children with asthma to those with other allergic diseases following SARS-CoV-2 infection. The study did not include an uninfected asthma comparator or an asthma-anchored longitudinal pre–post analysis. Outcomes were primarily symptom-based (e.g., cough, wheeze, expectoration, long COVID) rather than validated post-acute asthma outcomes, and asthma control was not assessed using validated instruments (ACT, cACT, ACQ, or GINA control), nor was asthma exacerbation risk evaluated using standard clinical endpoints. |
| Ren J, Pang W, Luo Y, et al. Impact of allergic rhinitis and asthma on COVID-19 infection, hospitalization, and mortality. Journal of Allergy and Clinical Immunology: In Practice. 2022;10:124–133. | Large population-based cohort study (UK Biobank) evaluating SARS-CoV-2 infection risk, COVID-19 hospitalization, and mortality in individuals with allergic rhinitis and/or asthma, with asthma analysed as a baseline exposure/risk factor. The study did not assess post-acute (≥4 weeks) asthma-specific outcomes following SARS-CoV-2 infection, such as asthma control, exacerbation risk, medication changes, or longitudinal asthma trajectory, and therefore did not meet the review’s outcome and timing eligibility criteria. |
| Robinson LB, Fu X, Bassett IV, et al. COVID-19 severity in hospitalized patients with asthma: A matched cohort study. Journal of Allergy and Clinical Immunology: In Practice. 2021;9(1):497–500. | Matched cohort study restricted to hospitalised patients with acute SARS-CoV-2 infection, evaluating COVID-19 severity outcomes (ICU admission, mechanical ventilation, in-hospital mortality) with asthma analysed as a baseline exposure/risk factor. The study did not assess post-acute (≥4 weeks) asthma-specific outcomes following recovery, such as asthma control, post-COVID exacerbation risk, medication changes, or longitudinal asthma trajectory, and therefore did not meet the review’s outcome timing and scope eligibility criteria. |
| Sansone F, Di Filippo P, Russo D, et al. Lung function assessment in children with Long-Covid syndrome. Pediatric Pulmonology. 2024;59:472–481. | Prospective pediatric observational study evaluating lung function parameters, fractional exhaled nitric oxide (FeNO), and lung ultrasound findings in children with Long-COVID syndrome, irrespective of asthma status. Asthma was analysed only as a comorbidity/covariate, and the study did not assess asthma-specific post-acute outcomes, such as asthma control (ACT/cACT/ACQ), asthma exacerbation risk, asthma medication changes, or asthma-related healthcare utilisation following SARS-CoV-2 infection. The primary focus was Long-COVID–related respiratory physiology, rather than post-acute asthma outcomes. |
| Shah SA, Quint JK, Nwaru BI, Sheikh A. Impact of COVID-19 pandemic on asthma exacerbations: Retrospective cohort study of over 500,000 patients in a national English primary care database. The Lancet Regional Health – Europe. 2022;19:100428. | Large national retrospective time-series cohort study evaluating changes in asthma exacerbation rates across pandemic periods (2020–2021) compared with pre-pandemic calendar years (2016–2019). The study did not classify individuals by confirmed SARS-CoV-2 infection status nor assess post-acute (≥4 weeks) asthma outcomes following infection using infected–uninfected or within-person pre–post infection comparisons. Observed effects primarily reflect pandemic-level public health measures and reduced circulation of respiratory viruses, rather than the post-COVID asthma disease trajectory targeted in this review. |
| Siu KK, Yu MKL, Rosa Duque JS, Chan SHS, Lau YL, Lee SL. Population-based study on hospital admissions for pediatric status asthmaticus: from before to after the COVID-19 pandemic. Frontiers in Pediatrics. 2025. | Population-based ecological analysis comparing status asthmaticus admissions across calendar periods (before, during, and after the COVID-19 pandemic) without individual-level classification by confirmed SARS-CoV-2 infection. The study evaluates pandemic-period and public-health–measure effects on pediatric hospitalisations rather than post-acute (≥4 weeks) asthma control or exacerbation outcomes following SARS-CoV-2 infection in a predefined asthma cohort, and lacks infection-anchored comparators or within-patient pre–post analyses relevant to this review. |
| Smith VA, Berkowitz TSZ, Hebert P, Wong ES, Niederhausen M, Berry K, et al. Design and analysis of outcomes following SARS-CoV-2 infection in Veterans. BMC Medical Research Methodology. 2023;23:81. | Methodological study describing the design and matching strategy used to construct cohorts of SARS-CoV-2–infected and uninfected Veterans for subsequent outcome analyses. The article does not report primary clinical results and does not evaluate asthma-specific post-acute outcomes, such as asthma control or asthma exacerbation risk following SARS-CoV-2 infection, which were the primary outcomes of interest for this review. |
| Svist PG, Torchinsky NV, Avdeev SN, Briko NI. Comparative assessment of the quality of life in patients with bronchial asthma and chronic obstructive pulmonary disease before and after COVID-19. Perm Medical Journal. 2024;41(5):19–26. | Retrospective comparative study assessing generic quality-of-life outcomes (EuroQol-5D and visual analogue scale) before and after COVID-19 infection in patients with asthma and COPD. The study did not evaluate asthma-specific post-acute outcomes, such as validated asthma control measures (ACT, cACT, ACQ), asthma exacerbation risk, medication changes, or asthma-related healthcare utilisation, and therefore did not meet the review’s outcome eligibility criteria. |
| Tichopád A, Žigmond J, Jeseňák M, et al. Adherence to application technique of inhaled corticosteroid in patients with asthma and COVID-19 improves outcomes. BMJ Open Respiratory Research. 2024;11:e001874. | Assessed the association between inhaled corticosteroid adherence and COVID-19 severity, hospitalisation, lung function changes, and quality of life, treating asthma as a baseline condition and effect modifier. The study focused on acute and recovery-phase COVID-19 outcomes rather than post-acute (≥4 weeks) asthma-specific outcomes, such as asthma control or post-infection exacerbation risk following SARS-CoV-2 infection, and therefore did not meet the outcome and timing eligibility criteria of this review. |
| Turdikhodjaevna KB, Tashmatova GA. Clinical and laboratory features and their correlation in children with bronchial asthma in the post-COVID period. International Journal of Medical Science and Public Health Research. 2025;6(9):46–52. | Cross-sectional observational study primarily examining correlations between laboratory and immunological parameters (macro- and micronutrients, IgE, eosinophils, apoptotic markers) and asthma severity categories. The study did not assess post-acute (≥4 weeks) asthma control using validated instruments (ACT, cACT, or ACQ), did not evaluate asthma exacerbation risk using clinical endpoints (e.g., systemic corticosteroid use, emergency department visits, or hospitalisation), and did not perform longitudinal pre–post or comparator-based analyses of post-SARS-CoV-2 asthma outcomes. |
| Verduri A, Hewitt J, Carter B, Tonelli R, Clini E, Beghè B. Prevalence of asthma and COPD in a cohort of patients at the follow up after COVID-19 pneumonia. Pulmonology. 2023;29:247–249. | Evaluated the prevalence of asthma and COPD and post-COVID respiratory functional outcomes (dyspnoea, spirometry, DLCO, exercise tolerance) in a cohort of patients recovering from COVID-19 pneumonia, rather than assessing post-acute (≥4 weeks) asthma-specific outcomes (e.g., asthma control or exacerbation risk) following SARS-CoV-2 infection in a predefined asthma population. The study lacked asthma-anchored comparators or longitudinal pre–post analyses relevant to the objectives of this review. |
| Vivaldi G, Talaei M, Pfeffer PE, Shaheen SO, Martineau AR. COVID-19 severity and risk of SARS-CoV-2-associated asthma exacerbation by time since booster vaccination: a longitudinal analysis of data from the COVIDENCE UK study. BMJ Open Respiratory Research. 2025;12:e003158. | Prospective longitudinal cohort study examining the association between time since COVID-19 booster vaccination and acute SARS-CoV-2 infection characteristics, including infection-associated asthma exacerbations occurring during or immediately following breakthrough infection. The study focused on acute, infection-triggered exacerbations and vaccination effects, and did not assess post-acute (≥4 weeks) asthma outcomes or longer-term asthma disease trajectory following recovery from SARS-CoV-2 infection, which were the primary outcomes of interest for this review. |
| Wang L, Foer D, Zhang Y, Karlson EW, Bates DW, Zhou L, et al. Post–acute COVID-19 respiratory symptoms in patients with asthma: an electronic health records–based study. Journal of Allergy and Clinical Immunology: In Practice. 2023;11:825–835. | Retrospective EHR-based cohort study evaluating post-acute respiratory symptom burden (e.g., dyspnoea, cough, wheeze, bronchospasm) following SARS-CoV-2 infection in patients with asthma compared with those without asthma. The study focused on symptom prevalence and incident respiratory symptoms identified from clinical notes and did not assess asthma-specific post-acute outcomes, such as asthma control, asthma exacerbation risk, or changes in asthma management, which were the primary outcomes of interest for this review. |
| Zo S, Kim Y, Kim JS, Kang MG, Moon JY, Park J, et al. Risk of Stroke in COVID-19 Survivors With Asthma: A Nationwide, Population-Based Cohort Study. Journal of Korean Medical Science. 2025;40:e247. | Nationwide population-based cohort study evaluating incident stroke risk following SARS-CoV-2 infection in individuals with asthma, stratified by COVID-19 severity and compared with the general population. The study focused on neurological and cardiovascular outcomes after COVID-19 recovery and did not assess post-acute (≥4 weeks) asthma-specific outcomes, such as asthma control or asthma exacerbation risk following SARS-CoV-2 infection, which were the primary outcomes of interest for this review. |
| Zo S, Lee H, Jeong CY, Kim BG, Chung JE, Kim Y, et al. COVID-19 and long-term risk of ischemic heart disease in asthma. Allergy Asthma Immunology Research. 2025;17:135–146. | Nationwide population-based cohort study evaluating the long-term risk of ischemic heart disease following SARS-CoV-2 infection in individuals with asthma, stratified by COVID-19 severity. The study focused on cardiovascular outcomes after COVID-19 recovery and did not assess post-acute (≥4 weeks) asthma-specific outcomes, such as asthma control or asthma exacerbation risk following SARS-CoV-2 infection, which were the primary outcomes of interest in this review. |
| Árquez-Mendoza M, Franco-Valencia K, Anaya-Romero M, Acevedo-Cerchiaro M, Fragozo-Messino S, Pertuz-Guzman DL, Luna-Carrascal J. Asthma Hospitalizations in Children Before and After COVID-19: Insights from Northern Colombia. Clinics and Practice. 2025;15:184. | Evaluated pediatric asthma hospitalizations and severity across pre-pandemic, pandemic, and post-pandemic calendar periods without individual-level classification by confirmed SARS-CoV-2 infection. Outcomes reflect acute exacerbation severity and healthcare utilisation rather than post-acute (≥4 weeks) asthma outcomes following confirmed infection, and comparators are time-period–based rather than infected vs uninfected asthma patients or within-patient pre–post infection analyses. |
| Özerden Özcan S, Polat Terece S, Yalçın G, Ertoy Karagöl Hİ, Bakırtaş A. The effect of the COVID-19 pandemic on long-term treatment compliance and disease control in children with persistent asthma. The Turkish Journal of Pediatrics. 2023;65(5):739–747. | Pandemic-period observational study evaluating asthma treatment compliance, exacerbations, and asthma control before versus during the COVID-19 pandemic, rather than assessing post-acute (≥4 weeks) asthma outcomes following confirmed SARS-CoV-2 infection. Asthma outcomes were analysed in relation to pandemic-related factors (e.g., reduced upper respiratory tract infections, school attendance, behavioural changes) and not anchored to individual SARS-CoV-2 infection or recovery, and therefore did not meet the review’s exposure and timing eligibility criteria. |
| Öztürk GK, Böncüoğlu E, Kıymet E, Şahinkaya Ş, Cem E, Yılmaz Çelebi M, et al. Long-term follow-up of children after COVID-19 infection and monitoring pulmonary functions. Pediatrics International. 2025;67:e70274. | Prospective cohort study evaluating long-term respiratory symptoms and pulmonary function test changes in children following SARS-CoV-2 infection. The study population was not restricted to children with pre-existing asthma; asthma was reported only as a comorbidity or as new-onset asthma diagnosed after COVID-19 infection. Outcomes focused on spirometric parameters and persistent respiratory complaints rather than asthma-specific post-acute outcomes. The study did not assess asthma control using validated instruments (ACT, cACT, or ACQ), did not evaluate asthma exacerbation risk, and lacked an asthma-anchored comparator or longitudinal pre–post assessment of asthma outcomes. |
