# Supplementary material for: Asthma control and exacerbation risk following SARS-CoV-2 infection in the post-acute COVID-19 phase: a systematic review
Source: Allergy Asthma Clin Immunol. 2026 Apr 15;22:36. doi: 10.1186/s13223-026-01027-z (PMC13220527; doi:10.1186/s13223-026-01027-z)
Supplement: Supplementary file 3 — Supplementary Material 3. [file 13223_2026_1027_MOESM3_ESM.docx]

Supplementary Table S3. Newcastle–Ottawa Scale (NOS) quality assessment of included cohort and case–control studies.

| Author, year | Selection (0–4) | Comparability (0–2) | Outcome (0–3) | Total (0–9) | Overall risk of bias | Notes |
| --- | --- | --- | --- | --- | --- | --- |
| Abdul-Razzak (2025) [[21](#_ENREF_21)] | 3 | 1 | 2 | 6 | Moderate | Single-centre retrospective paediatric cohort with clear definition of asthma and SARS-CoV-2 infection (PCR/RAT). Included infected and uninfected asthma comparators with before–after measurements. Outcomes (GINA asthma control, exacerbation frequency, FeNO, spirometry) were clinically relevant but largely based on routine clinical records. No multivariable adjustment for confounding; analyses were predominantly unadjusted. Follow-up adequate for post-acute outcomes, but outcome assessment not blinded. |
| Abdul-Razzak (2025) [[20](#_ENREF_20)] | 3 | 1 | 2 | 6 | Moderate | Single-centre retrospective paediatric cohort; clear case definition of asthma and SARS-CoV-2 infection; comparator group included. Limited adjustment for confounding and no multivariable analysis; outcomes based on clinical records and follow-up visits without blinded assessment. |
| Agondi (2022) [[22](#_ENREF_22)] | 3 | 1 | 2 | 6 | Moderate | Single-centre retrospective adult cohort from a tertiary asthma clinic; clear case definition of asthma and laboratory-confirmed SARS-CoV-2 infection; comparator group included. Limited control for confounding with no multivariable adjustment; outcomes based on ACT change and treatment escalation without blinded assessment. |
| Chang (2024) [[23](#_ENREF_23)] | 3 | 1 | 2 | 6 | Moderate | Prospective case–control study of asthmatic children with and without SARS-CoV-2 infection. Asthma diagnosis established by pediatric respiratory specialists; exposure confirmed by rapid antigen testing. Comparator group of uninfected asthmatic children included. Asthma control assessed using a validated instrument (cACT), with objective secondary outcomes (FeNO, spirometry). Multivariable adjustment performed for key covariates; however, single-centre design, short post-acute follow-up (~1 month), and absence of longitudinal outcome assessment limit comparability and outcome robustness. |
| Choi (2025) [[24](#_ENREF_24)] | 4 | 2 | 2 | 8 | Low | Nationwide retrospective cohort study using the Korean HIRA database. Large, population-based asthma cohort with near-complete coverage. SARS-CoV-2 infection and asthma identified using validated ICD-10 codes. Within-person pre–post comparison (12 months before vs 12 months after infection) with recurrent-event modelling. Extensive sensitivity analyses excluding early events (≤14 days) and excluding patients with COPD. Limitations include claims-based outcome definitions and lack of clinical asthma control measures or lung function data. |
| Duong (2025) [[25](#_ENREF_25)] | 4 | 2 | 3 | 9 | Low | Large matched retrospective cohort; PCR-confirmed exposure; recurrent-event modelling with extensive confounder adjustment. |
| Gaietto (2025) [[27](#_ENREF_27)] | 4 | 1 | 2 | 7 | Moderate | Prospective registry-based pediatric cohort with clear definition of asthma and laboratory-confirmed SARS-CoV-2 infection. Within-person pre–post comparison using validated asthma control instruments (ACT/cACT) and objective spirometry outcomes. Multivariable mixed-effects models adjusted for age, sex, race, and baseline asthma severity. Single-centre design and reliance on routine clinical follow-up may introduce selection bias; post-acute asthma exacerbations were not assessed. |
| Gaietto (2023) [[26](#_ENREF_26)] | 4 | 2 | 2 | 8 | Low | Prospective registry-based pediatric case–control study with clear definition of asthma and laboratory-confirmed SARS-CoV-2 infection. Included an uninfected asthma control group and within-person pre–post comparisons. Asthma control assessed using validated instruments (ACT/cACT), with objective spirometry outcomes. Multivariable analyses adjusted for key confounders (age, sex, race, baseline asthma severity, and time to follow-up). Single-centre design and reliance on routine EHR data may introduce selection bias; post-acute asthma exacerbations were not evaluated as outcomes. |
| Kwok (2023) [[28](#_ENREF_28)] | 3 | 2 | 2 | 7 | Moderate | Single-centre case–control study of adults with physician-diagnosed asthma; clear ascertainment of SARS-CoV-2 infection (RT-PCR/RAT) and validated asthma control outcome (ACT). Comparator group matched on key demographic and clinical variables with multivariable adjustment for major confounders. Outcomes partly based on retrospective clinical records; exacerbation outcomes analysed descriptively and using unadjusted models only; follow-up duration moderate. |
| Kwok (2025) [[29](#_ENREF_29)] | 3 | 2 | 2 | 7 | Moderate | Prospective follow-up cohort of adults with physician-diagnosed asthma from a tertiary asthma clinic; clear ascertainment of SARS-CoV-2 infection (PCR/RAT) and outcome assessment using ACT and clinically defined exacerbations. Multivariable adjustment for key confounders performed. Single-centre design and modest sample size limit representativeness; outcome assessment not blinded. |
| Lee (2024) [[30](#_ENREF_30)] | 4 | 2 | 3 | 9 | Low | Nationwide population-based cohort; RT-PCR–confirmed SARS-CoV-2 exposure; well-defined asthma population; 1:1 propensity score matching with extensive covariate adjustment; objective outcome definitions; adequate follow-up. |

Abbreviations: ACT, Asthma Control Test; cACT, Childhood Asthma Control Test; COPD, chronic obstructive pulmonary disease; EHR, electronic health record; FeNO, fractional exhaled nitric oxide; GINA, Global Initiative for Asthma; HIRA, Health Insurance Review and Assessment Service; NOS, Newcastle–Ottawa Scale; PCR, polymerase chain reaction; RAT, rapid antigen test; RT-PCR, reverse transcription polymerase chain reaction; SARS-CoV-2, severe acute respiratory syndrome coronavirus 2.
